# Macroscopic phase resetting-curves determine oscillatory coherence and signal transfer in inter-coupled neural circuits

**DOI:** 10.1371/journal.pcbi.1007019

**Published:** 2019-05-09

**Authors:** Grégory Dumont, Boris Gutkin

**Affiliations:** 1 Group for Neural Theory, LNC INSERM U960, DEC, Ecole Normale Supérieure PSL* University, Paris, France; 2 Center for Cognition and Decision Making, Institute for Cognitive Neuroscience, NRU Higher School of Economics, Moscow, Russia; Ghent University, BELGIUM

## Abstract

Macroscopic oscillations of different brain regions show multiple phase relationships that are persistent across time and have been implicated in routing information. While multiple cellular mechanisms influence the network oscillatory dynamics and structure the macroscopic firing motifs, one of the key questions is to identify the biophysical neuronal and synaptic properties that permit such motifs to arise. A second important issue is how the different neural activity coherence states determine the communication between the neural circuits. Here we analyse the emergence of phase-locking within bidirectionally delayed-coupled spiking circuits in which global gamma band oscillations arise from synaptic coupling among largely excitable neurons. We consider both the interneuronal (ING) and the pyramidal-interneuronal (PING) population gamma rhythms and the inter coupling targeting the pyramidal or the inhibitory neurons. Using a mean-field approach together with an exact reduction method, we reduce each spiking network to a low dimensional nonlinear system and derive the macroscopic phase resetting-curves (mPRCs) that determine how the phase of the global oscillation responds to incoming perturbations. This is made possible by the use of the quadratic integrate-and-fire model together with a Lorentzian distribution of the bias current. Depending on the type of gamma (PING vs. ING), we show that incoming excitatory inputs can either speed up the macroscopic oscillation (phase advance; type I PRC) or induce both a phase advance and a delay (type II PRC). From there we determine the structure of macroscopic coherence states (phase-locking) of two weakly synaptically-coupled networks. To do so we derive a phase equation for the coupled system which links the synaptic mechanisms to the coherence states of the system. We show that a synaptic transmission delay is a necessary condition for symmetry breaking, i.e. a non-symmetric phase lag between the macroscopic oscillations. This potentially provides an explanation to the experimentally observed variety of gamma phase-locking modes. Our analysis further shows that symmetry-broken coherence states can lead to a preferred direction of signal transfer between the oscillatory networks where this directionality also depends on the timing of the signal. Hence we suggest a causal theory for oscillatory modulation of functional connectivity between cortical circuits.

## Author summary

Large scale brain oscillations emerge from synaptic interactions within neuronal circuits. Over the past years, such macroscopic rhythms have been suggested to play a crucial role in routing the flow of information across cortical regions, resulting in a functional connectome. The underlying mechanism is cortical oscillations that bind together following a well-known motif called phase-locking. While there is significant experimental support for multiple phase-locking modes in the brain, it is still unclear what is the underlying mechanism that permits macroscopic rhythms to phase lock. In the present paper we take up with this issue, and to show that, one can study the emergent macroscopic phase-locking within the mathematical framework of weakly coupled oscillators. We find that under synaptic delays, fully symmetrically coupled networks can display symmetry-broken states of activity, where one network starts to lead in phase the second (also sometimes known as stuttering states). When we analyse how incoming transient signals affect the coupled system, we find that in the symmetry-broken state, the effect depends strongly on which network is targeted (the leader or the follower) as well as the timing of the input. Hence we show how the dynamics of the emergent phase-locked activity imposes a functional directionality on how signals are processed. We thus offer clarification on the synaptic and circuit properties responsible for the emergence of multiple phase-locking patterns and provide support for its functional implication in information transfer.

## Introduction

Ranging from infraslow to ultrafast, brain rhythms are a nearly omni-present phenomenon covering more than four orders of magnitude in frequency. Of this variety of rhythms, gamma oscillations, falling in the frequency band of 30–150 Hz, is arguably the most studied rhythmic brain activity pattern [1, 2]. Coherent gamma oscillations have been reported in many brain regions, across many species, and is associated with a variety of cognitive tasks [3, 4]. There is nowadays growing evidence that the gamma cycle results from emergent dynamics of cortical networks, as a natural consequence of the interplay between interconnected pyramidal cells and subnetworks of interneurons [5, 6].

Although brain rhythms such as gamma oscillations emerge locally [6], they are known to interact in a coherent fashion across the cortical scale [7, 8]. As such, macroscopic oscillations within different brain regions show multiple phase relationships that are persistent across time [9]. Such cross-coupling is crucial for a recently developed theory of how oscillations shape the information transfer within and across the cortex, the communication through coherence (CTC) hypothesis, it is further believed to be implicated in a number of higher cognitive functions. For example, enhanced inter-areal gamma-band coherence is considered as the neural correlate of selective attention, in which a network receiving several informational stimulus can preferentially react to one or another depending on task relevance [7].

The CTC hypothesis proposes a mechanism by which gamma rhythms may regulate the information flow [2]. The rationale behind it is that gamma oscillations are the consequence of rhythmic inhibitory feedback inducing an hyperpolarization of the principle cell membrane potential [5, 6]. Synaptic inputs targeting excitatory cells are then expected to cause a stronger reaction when the inhibition drops off. This gives rise to a temporal window of excitability within the oscillatory cycle during which pyramidal neurons are more likely to respond to stimulation [10]. Ongoing oscillatory firing patterns rhythmically modulate the excitability of networks, and therefore, two oscillating neural groups communicate more efficiently when they maintain a coherent relationship: they can consecutively send their information at the most excitable phase of the target network [4, 7].

According to the CTC hypothesis, neuronal interactions and transfer of information are dynamically shaped by the phase relationship between neuronal oscillations [11]. In fact it has been proposed that macroscopic rhythms offer a way of adjusting the effectivity of functional connectivity while leaving untouched the anatomical connections [9] and resulting in a functional connectivity [12, 13]. This functional connectivity, often defined in correlational or information transmission terms, is determined by the relative phase relationship between the communicating networks. Note that an optimal locking mode is not always at the zero phase lag or perfect spike synchrony (or macroscopic synchrony, as we will see in this manuscript). The reason is that, spike transmission from one network to another is not instantaneous and, depending on the distance, projection across the brain can take up to hundreds of milliseconds [14]. Therefore oscillations should be lagged in order to see their spikes arriving at the most excitable phase. This most excitable phase also depends on the biophysical properties of the constituent neurons and of the emergent rhythms (e.g. as characterized by the network-wide phase response curves [15]). An optimal phase difference will thus depend on the properties of the neural groups at work and the distance between the two [16, 17]. Recent experimental studies have reported a multiplicity of phase differences and it has been argued that such a diversity might facilitate information selectivity [17]. In other words, the emergent collective dynamics of the coupled networks defines how information is chaneled between them, hence by controlling these dynamics one can control dynamically the flow of information without having to change the structural connectivity.

Over the past few years, computational studies have devoted a great deal of attention to uncovering the precise functional roles of gamma patterns and gamma interaction. Doing so, they have been able to reproduce experimental findings in support of several predictions of the CTC hypothesis. For instance, modeling approaches have shown that the gamma cycle generates a temporal window of excitability [18], which is suitable to suppress irrelevant stimuli [19, 20]. Others studies have demonstrated that the mutual information between two neural groups engaged in rhythmic patterns is tuned with respect to their phase lag [21, 22], and a directionality in the flow of information emerges through a symmetry breaking in the phase relationship [12, 13]. A diversity of phase lags can then be observed which benefits information coding and stimulus reconstruction [23]. Finally, in a rather different line of thinking from the main current view of CTC, computational studies have exposed how cortical oscillations could implement a multiplexing [24–26].

However, the underlying mechanisms responsible for the emergence of the multiple phase-locking modes and of the ensuing functional connectivity as proposed by the CTC are not trivial. So far, no mechanistic view to explain the observed variety of phase lags has been proposed. The question is then to identify through what synaptic mechanisms can these rhythms coordinate their temporal relationships in such a diversity of locking modes. Answering this question is crucial and knowing the chain of causation that allows for coherent oscillations is key to understanding their functional role [27, 28]. Hence, a subsequent question is how one can characterize the functional connectivity associated with the various phase-locking modes and how directed signal transmission can ensue.

Here we approach the questions above by studying analytically the dynamical emergence of phase-locking within two bidirectionally delayed-coupled gamma-oscillatory spiking networks. Importantly, the neurons within the circuits have a relatively wide distribution of intrinsic excitability, meaning that most of them are not intrinsically oscillating. Hence the gamma rhythm in our network is an emergent property of the global dynamics, as opposed to phase synchrony of coupled oscillators (see [29] for instance). Furthermore, the design of the interconnections between our networks is inspired from previous research [13, 21, 22] to essentially capture multiple communicating brain regions where transfer of information takes place. Each network is assumed to be made up of pyramidal cells and interneurons, and each cell is characterized by a conductance-based neural model [30, 31]. A synaptic delay is included to account for possible long range distances separating the circuits [14]. We then take advantage of a thermodynamic approach combined with a reduction theory to simplify each network description—see [32–34]—and to express the macroscopic phase resetting curve (mPRC) of their oscillatory cycle [15, 35, 36].

The network mPRC is an important causal measure which allows us to use the weakly coupled oscillator theory [37, 38] to characterize the inter-network dynamics. The fundamental assumption at the core of this theoretical setting is that synaptic projections from one circuit to another must be sufficiently weak. Please note that the weak coupling condition is not on the synaptic connections within each of the circuits, but only across them. The weak coupling condition allows one to take advantage of a variety of mathematical techniques and to abbreviate the bidirectionally delayed-coupled spiking circuits description to a single phase equation [39, 40]. Note that the study of delayed coupled oscillators has already received some attention in computational neuroscience [41–44]. This simplification significantly reduces the complexity of the interacting macroscopic oscillations, making them mathematically tractable, while at the same time capturing crucial principles of phase-locking.

As we show below, an analysis of the phase equation sheds light on the synaptic mechanism enabling circuits with emergent global oscillations to bind together. We give particular attention to the central role played by the synaptic conduction delays in producing symmetry-broken states of activity (with purely symmetric connectivity), i.e to permit the emergence of a variety of non-symmetric phase lags. In other word, we look for conditions under which the role played by the two networks is not symmetric anymore: one network leads the dynamics and the other one follows. Such a collection of phase lags has been suggested to facilitate the control and selection of the information flow through anatomical pathways [17], and conduction delays have been at the core of recent discussions regarding the CTC hypothesis [45]. Our final goal is then to show that non-symmetric phase lags lead to a directed functional coupling between the networks. We indeed show that symmetry-broken states induce a preferred direction of signal transfer between the networks, and therefore provide theoretical support for the role of oscillations in modulating functional connectivity between cortical circuits [12, 13].

The paper is structured as follows. First, we present the network and neural model which will be used throughout. We explain the low dimensional system for which we can perform a bifurcation analysis and extract the infinitesimal PRC. From there, we compute the so-called interaction function and reduce the bidirectionally delayed-coupled spiking networks to a unique phase equation. The analysis of the phase equation enables us to make several predictions on the locking states between the emerging oscillations. We support our theoretical findings with extensive numerical illustrations and discuss our results in light of the CTC hypothesis and functional connectivity. Finally, the mathematical techniques are explained in a detailed Methods section at the end of the paper.

## Results

### The network and its reduced description

Our generic cortical circuit is assumed to be made up of *N*_*e*_ excitatory cells (E-cells) and *N*_*i*_ inhibitory cells (I-cells) coupled in an all-to-all fashion. Each cell is described by a well-established model—the quadratic integrate-and-fire (QIF), see [46]—which is known to capture the essential dynamical features of the neural voltage [30]. The action potential is taken into account by a discontinuous reset mechanism (note that for the QIF this reset is not at the firing threshold as for the regular integrate and fire model, but either at the top of during the active phase of the action potential). Whenever a cut off value *v*_*th*_ is reached, the voltage is instantaneously set to *v*_*r*_, a reset parameter. To permit analytical computations, we use the canonical form of the QIF that corresponds to the normal form for the saddle-node on an invariant cycle bifurcation, where the threshold *v*_*th*_ and reset *v*_*r*_ are respectively taken at plus and minus infinity [30]. The QIF reads

$$\begin{array}{c} \tau \frac{d}{dt}v_{j}=\eta_{j}+v_{j}^{2}+I, \end{array}$$ (1)

where *v*(*t*) is the neural voltage, *j* the neuron number, *τ* the membrane time constant, *η* the bias current that defines the intrinsic resting potential and firing threshold of the cell and finally *I*(*t*) the total synaptic current injected at the soma. To account for the network heterogeneity, the intrinsic parameter *η* is distributed randomly according to a Lorentzian distribution (Note that we choose this distribution form in order to facilitate our analysis):

$$\begin{array}{c} L\left(\eta \right)=\frac{1}{\pi }\frac{\Delta }{{\left(\eta -\bar{\eta }\right)}^{2}+\Delta^{2}}. \end{array}$$

Here $\bar{\eta }$ stands for the mean value (in the Cauchy sense) taken by the parameter *η* across the population and Δ is the half-width of the distribution. Note that the heavy-tailed Lorentzian distribution implies a wide range of intrinsic excitability, i.e. many neurons are not intrinsically oscillating and if they do, they have different firing frequency, as opposed to the classical framework of phasing of coupled oscillators (see [29] for instance). Indeed, when the external current *I*_*ext*_ is taken to be zero, the proportion of neurons not being intrinsic oscillator is given by

$$\begin{array}{c} \int_{-\infty }^{0}L\left(\eta \right)\;d\eta =\frac{1}{\pi }(\frac{\pi }{2}-\arctan(\frac{\bar{\eta }}{\Delta })), \end{array}$$

which can not be zero as soon as there is heterogeneity within the network. Note nonetheless that the proportion will be affected by the synaptic current.

The total synaptic current, *I*(*t*) is assumed to be the sum of an external input *I*^*ext*^(*t*) that takes into account inputs coming to the cell from sub-cortical structures or nearby cortical networks through lateral connections, and the synaptic inputs *s*_*e*_ and *s*_*i*_ which models the effect of recurrent connexions within the circuit, for the E-cells we have:

$$\begin{array}{c} I_{e}=I_{e}^{ext}+\tau_{e}s_{ee}-\tau_{e}s_{ei}, \end{array}$$

and for the I-cells:

$$\begin{array}{c} I_{i}=I_{i}^{ext}+\tau_{i}s_{ie}-\tau_{i}s_{ii}. \end{array}$$

The synaptic current, *s*(*t*), depends on the synapse type, for the excitatory synapse, for the E-cells, we have

$$\begin{array}{c} \tau_{s}\frac{d}{dt}s_{ee}=-s_{ee}+J_{ee}r_{e}, \end{array}$$

respectively for the inhibitory synapse,

$$\begin{array}{c} \tau_{s}\frac{d}{dt}s_{ei}=-s_{ei}+J_{ei}r_{i}, \end{array}$$

and of course for the I-cells

$$\begin{array}{c} \tau_{s}\frac{d}{dt}s_{ie}=-s_{ie}+J_{ie}r_{e}, \end{array}$$

respectively for the inhibitory synapse,

$$\begin{array}{c} \tau_{s}\frac{d}{dt}s_{ii}=-s_{ii}+J_{ii}r_{i}. \end{array}$$

**Fig 1:**
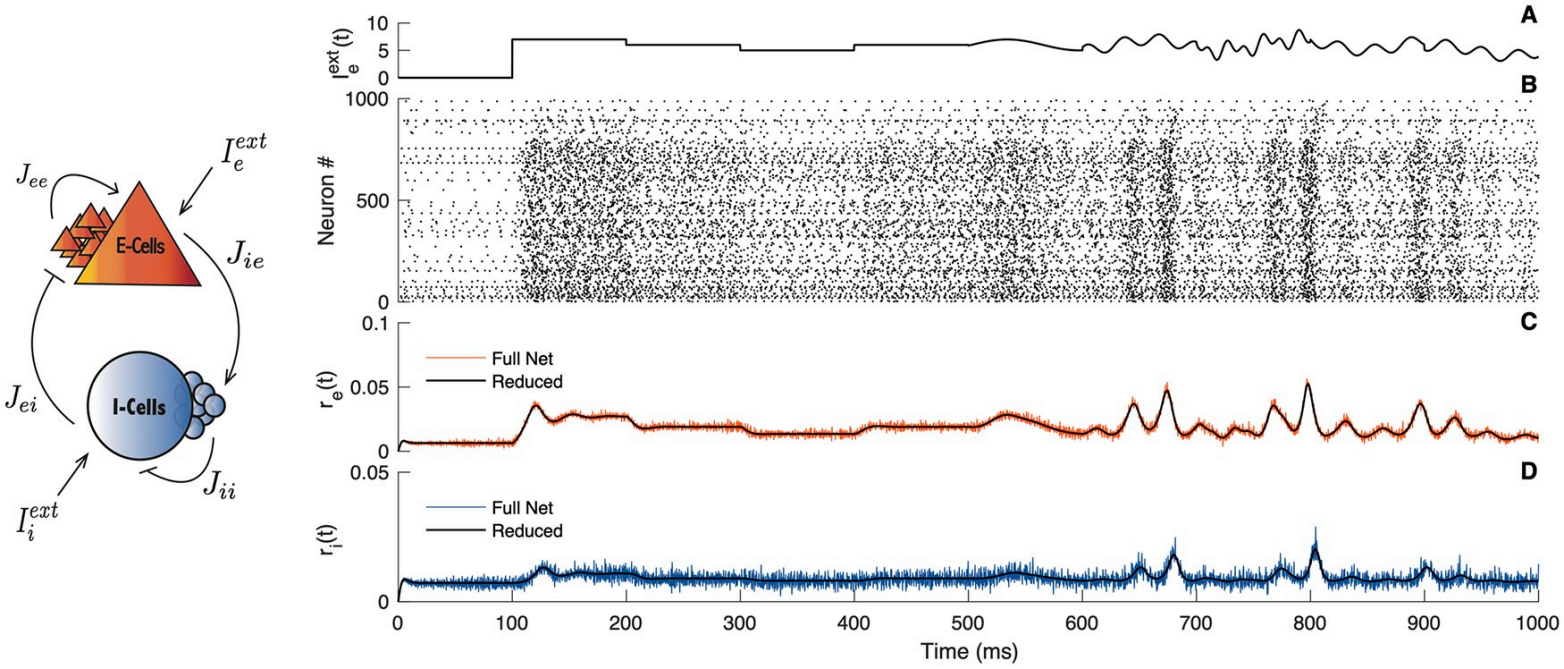
Comparison between the full network and the reduced system. Left panel: Schematic illustration of a canonical cortical neural network. The parameter *J*_*αβ*_ denotes the connectivity strength of the population *β* onto the population *α*. The external influence on the population *α* is denoted $I_{\alpha }^{ext}$. Right panels: A) Time evolution of the stimulus $I_{e}^{ext}$ on the E-cells. B) Spiking activity obtained from simulations of the full network, the first 800 cells are excitatory, the last 200 are inhibitory. C) Firing rate of the E-cells obtained from simulations of the full network (red line) compared with the reduced system (black line). D) Firing rate of the I-cells obtained from simulations of the full network (blue line) compared with the reduced system (black line). Parameters: *N*_*e*_ = *N*_*i*_ = 5000; Δ_*e*_ = Δ_*i*_ = 1; *τ*_*e*_ = *τ*_*i*_ = 10; *τ*_*se*_ = *τ*_*si*_ = 1; ${\bar{\eta }}_{e}={\bar{\eta }}_{i}=-5$; *J*_*ee*_ = 0; *J*_*ei*_ = 15; *J*_*ii*_ = 10; *J*_*ie*_ = 15; $I_{i}^{ext}=0$; *v*_*th*_ = 500; *v*_*r*_ = −500.

Here, *τ*_*s*_ the synaptic time constant, *J* the synaptic strength—see Fig 1—and *r*(*t*) the population firing rate. For the E-cells, we have:

$$\begin{array}{c} r_{e}\left(t\right)=\frac{1}{N_{e}}\sum_{k=1}^{N_{e}}\sum_{f}\delta \left(t-t_{f}^{k}\right), \end{array}$$

and for the I-cells, we have:

$$\begin{array}{c} r_{i}\left(t\right)=\frac{1}{N_{i}}\sum_{k=1}^{N_{i}}\sum_{f}\delta \left(t-t_{f}^{k}\right), \end{array}$$

where *δ* is the Dirac mass measure and $t_{f}^{k}$ are the firing time of the neuron numbered *k*.

To get a clear picture of how the synaptic structure shapes the firing patterns, we take advantage of a thermodynamic approach combined with a reduction method. The thermodynamic framework produces an average system written in terms of partial differential equations that is valid in the limit of an infinitely large number of neurons [47]. The reduction method allows further simplification and breaks down the mean-field system into a small set of differential equations [33, 34]. In our case, the low dimensional dynamical system reads (see Methods for more details of the derivation):

$$\begin{array}{c} \left\{ \begin{array}{cc} & \tau_{e}\frac{d}{dt}r_{e}=\frac{\Delta_{e}}{\pi \tau_{e}}+2r_{e}V_{e}\\ & \tau_{e}\frac{d}{dt}V_{e}=V_{e}^{2}+{\bar{\eta }}_{e}+I_{e}-\tau_{e}^{2}\pi^{2}r_{e}^{2},\\ & \tau_{s}\frac{d}{dt}s_{ee}=-s_{ee}+J_{ee}r_{e},\\ & \tau_{s}\frac{d}{dt}s_{ei}=-s_{ei}+J_{ei}r_{i}, \end{array}\right. \end{array}$$ (2)

and for the I-cells:

$$\begin{array}{c} \left\{ \begin{array}{cc} & \tau_{i}\frac{d}{dt}r_{i}=\frac{\Delta_{i}}{\pi \tau_{i}}+2r_{i}V_{i}\\ & \tau_{i}\frac{d}{dt}V_{i}=V_{i}^{2}+{\bar{\eta }}_{i}+I_{i}-\tau_{i}^{2}\pi^{2}r_{i}^{2}.\\ & \tau_{s}\frac{d}{dt}s_{ie}=-s_{ie}+J_{ie}r_{e},\\ & \tau_{s}\frac{d}{dt}s_{ii}=-s_{ii}+J_{ii}r_{i}, \end{array}\right. \end{array}$$ (3)

Here, *V*(*t*) represents the mean voltage (in the Cauchy sense) of the population, while *r*(*t*) still stands for the firing activity. Note that the two systems are coupled via the expression of the total current arriving on each sub-population:

$$\begin{array}{c} I_{e}=I_{e}^{ext}+\tau_{e}s_{ee}-\tau_{e}s_{ei}, \end{array}$$

and

$$\begin{array}{c} I_{i}=I_{i}^{ext}+\tau_{i}s_{ie}-\tau_{i}s_{ii}. \end{array}$$

The numerical simulations presented in Fig 1 compare the dynamics of the full network with the low dimensional system (2) and (3) in response to a continuous external stimulus. The time evolution of the external stimulus is seen in the first panel (Fig 1A), whereas the second panel gives the spiking activity obtained from a simulation of the full network (Fig 1B). In the subsequent panels (Fig 1C and 1D), the firing rate given by the reduced description is compared with the firing rate obtained from network simulations. We can see that both models are able to follow the stimulus amplitude in time (the time-interval averaged firing rate of spikes for the full system and the rate variable for the reduced version). The perfect agreement between the population activities convinced us that the reduced dynamical system captures the fundamental aspects of the population firing rate. In addition, such a reduced description provides an efficient way to carry out a study of the circuit since it can be simulated very quickly and it is amenable to mathematical analysis.

### Emerging rhythms and phase-resetting curve

To understand how the emergent network gamma oscillations can phase lock, it is essential to first consider their basic underlying mechanisms. To gain insights, we carried out a nonlinear analysis of the reduced system. This enabled us to reveal how the inhibitory feedback loop renders possible the emergence of macroscopic gamma rhythms. Two processes can be described: the PING and the ING [6].

**Fig 2:**
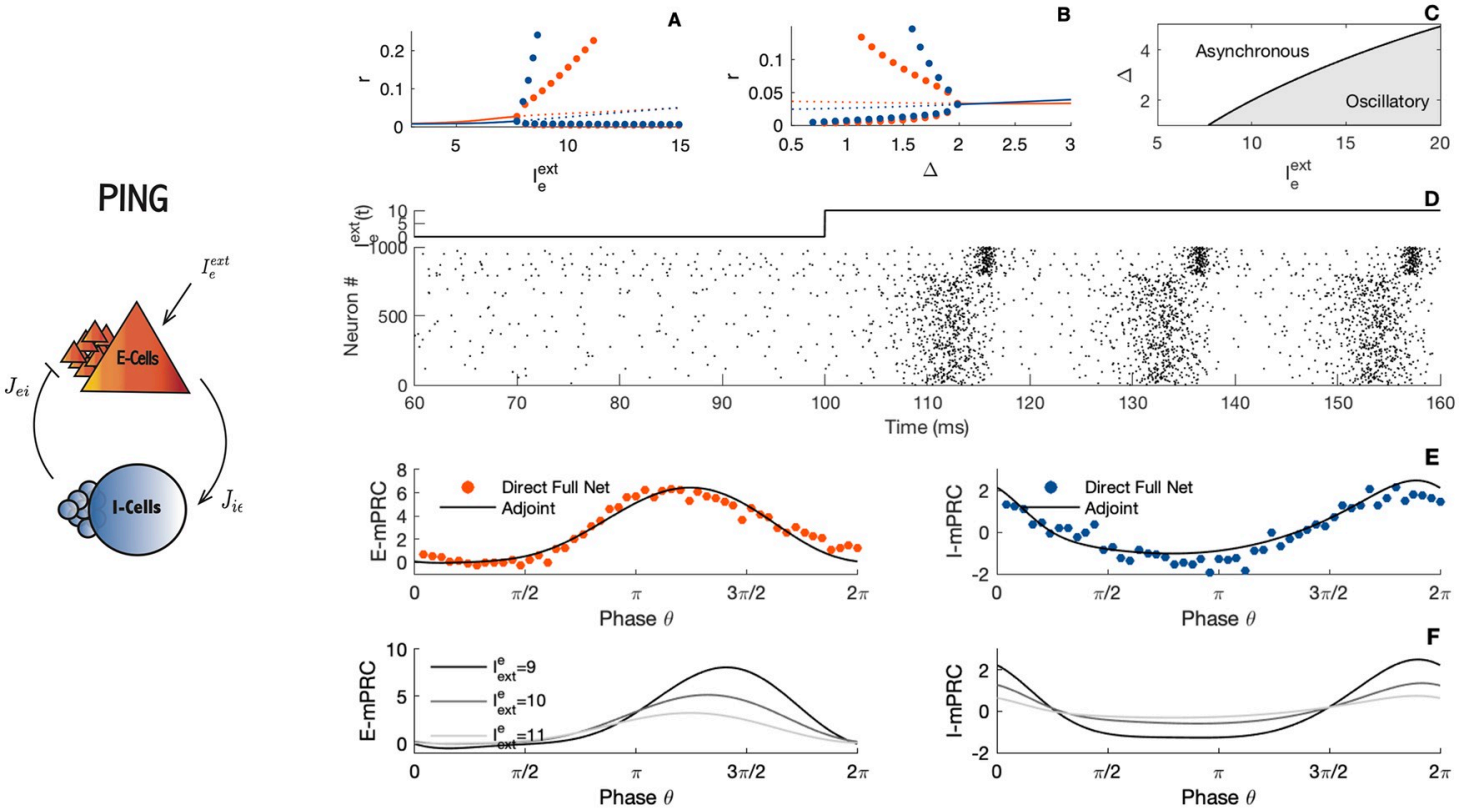
The PING interaction. Left panel: A schematic illustration of the PING (Pyramidal Interneuron Network Gamma) interaction. The parameters are as in Fig 1. Right panels: Nonlinear analysis of the PING rhythm. A-B) Bifurcation diagrams. The blue line, (respectively the red line), corresponds to the steady state of the inhibitory cells, (respectively the excitatory cells) while dots correspond to limit cycles. C) Stability region. D) The stimulus and corresponding raster plot of the spiking activity. E) Comparison between simulated and calculated mPRCs. The black line illustrates the analytical adjoint method while dots indicates direct perturbations of the full network. Red dots, perturbations are made on the E-cells, second row, with the blue dots, perturbations are made on the I-cells. F) PRC shape as a function of parameters obtained via the adjoint method for different values of the external current. Parameters: *N*_*e*_ = *N*_*i*_ = 5000; *τ*_*e*_ = *τ*_*i*_ = 10; *τ*_*se*_ = *τ*_*si*_ = 1; ${\bar{\eta }}_{e}={\bar{\eta }}_{i}=-5$; *J*_*ee*_ = 0; *J*_*ei*_ = 15; *J*_*ii*_ = 0; *J*_*ie*_ = 15; $I_{i}^{ext}=0$; *v*_*th*_ = 500; *v*_*r*_ = −500; panel A) Δ_*e*_ = Δ_*i*_ = 1; panel B) $I_{ext}^{e}=10$; panel D) Δ_*e*_ = Δ_*i*_ = 1; panel E-F) Δ_*e*_ = Δ_*i*_ = 1; $I_{ext}^{e}=10$, direct perturbations are made with a square wave current pulse (amplitude 5, duration 0.5).

In the PING (Pyramidal Interneuron Network Gamma) interaction, see Fig 2, the underlying synaptic machinery involves an interplay between the pyramidal cells and the inhibitory-spiking cells. For a chosen set of connectivity parameters, the dynamical system exhibits a Hopf bifurcation (Fig 2A), such that, enhancing the external stimulus upon the pyramidal cells induces a graded progression toward a coherent oscillatory regime. Note that this rhythmic regime disappears as the network heterogeneity is expanded (see Fig 2B and 2C). The rhythmic transition is illustrated with a simulation displayed in Fig 2D. A self-sustained oscillatory regime emerges as soon as the E-drive is strong enough. Of course, the presence of a Hopf bifurcation in the system should be put in relation with the seminal work of Wilson and Cowan [48], where a similar path to oscillations was found. Note that, in contrast to the Wilson-Cowan equation, the spiking network presented in here does not require excitatory-to-excitatory connection to oscillate.

**Fig 3:**
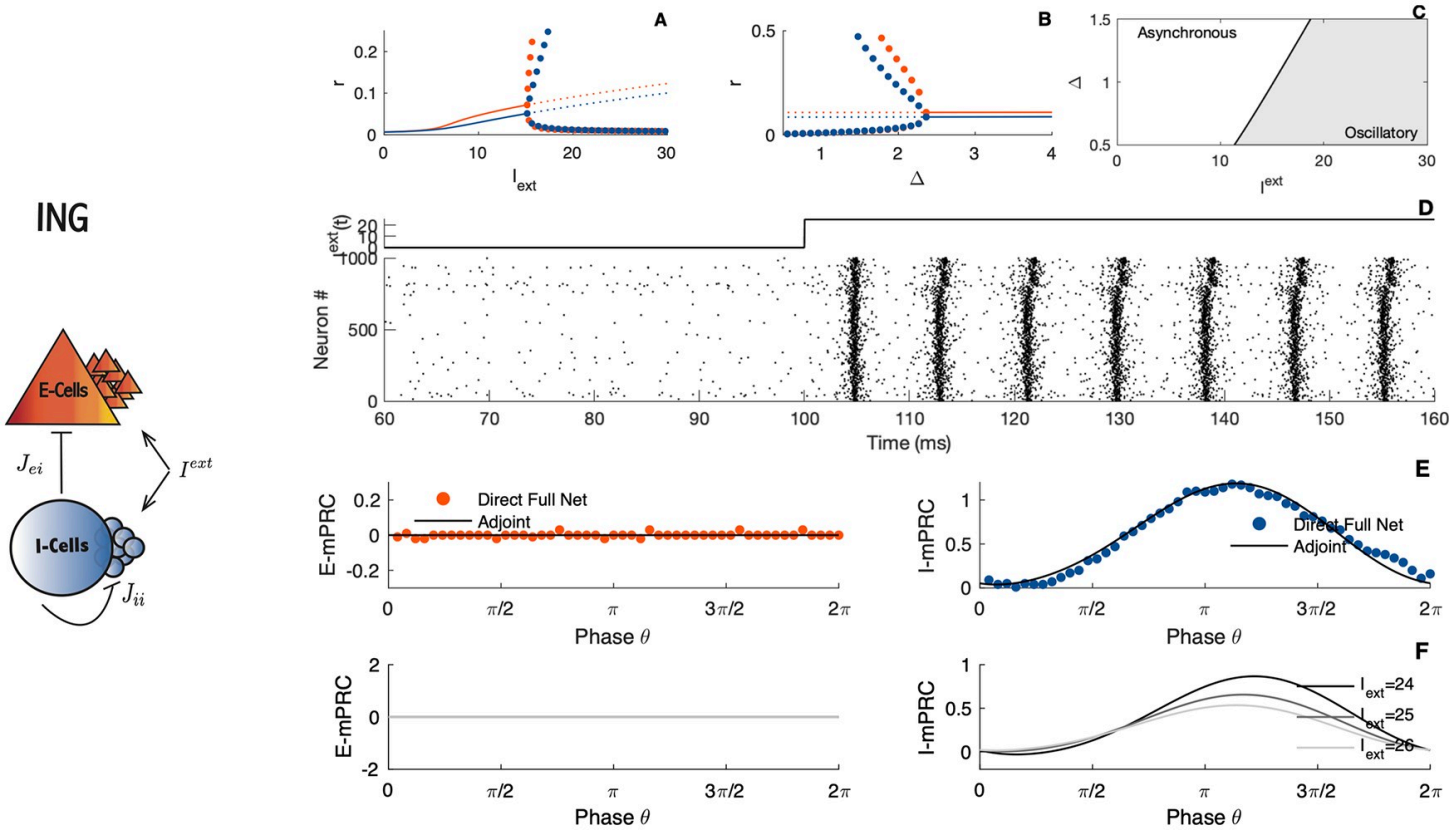
The ING interaction. Left panel: Schematic illustration of the ING (Interneuron Network Gamma) interaction. The parameters are as Fig 1, except that the extenal current *I*_*ext*_ goes on both populations. Right panels: Nonlinear analysis of the The ING interaction. A-B) Bifurcation diagrams. The blue line, (respectively the red line), corresponds to the steady state of the inhibitory cells, (respectively the excitatory cells) while dots correspond to limit cycles. C) Stability region. D) The stimulus and corresponding raster plot of the spiking activity. E) Comparison between simulated and calculated mPRCs. The black line illustrates the analytical adjoint method while dots indicates direct perturbations of the full network. Red dots, perturbations are made on the E-cells, second row, with the blue dots, perturbations are made on the I-cells. F) PRC shape as a function of parameters obtained via the adjoint method for different values of the external current. Parameters: *N*_*e*_ = *N*_*i*_ = 5000; *τ*_*e*_ = *τ*_*i*_ = 10; *τ*_*se*_ = *τ*_*si*_ = 1; ${\bar{\eta }}_{e}={\bar{\eta }}_{i}=-5$; *J*_*ee*_ = 0; *J*_*ei*_ = 10; *J*_*ii*_ = 15; *J*_*ie*_ = 0; *v*_*th*_ = 500; *v*_*r*_ = −500; panel A) Δ_*e*_ = Δ_*i*_ = 1; panel B) *I*_*ext*_ = 25; panel D) Δ_*e*_ = Δ_*i*_ = 1; panel E-F) Δ_*e*_ = Δ_*i*_ = 1; *I*_*ext*_ = 25, direct perturbations are made with a square wave current pulse (amplitude 5, duration 0.5).

In the ING (Interneuron Network Gamma) interaction, see Fig 3, the mechanism requires an inhibitory feedback from inhibitory-spiking cells onto themselves and the rhythm arises from this interconnected inhibitory network which in turn defines the excitatory spike times. The nonlinear analysis reveals a Hopf bifurcation as the external drive is raised (see Fig 3A). Again, this rhythmic regime disappears with too much heterogeneity (see Fig 3B and 3C). The network activity undergoes a transition from an asynchronous regime toward an oscillatory which is displayed in Fig 3D. Interestingly, the ING behavior can not emerge within the traditional rate equation proposed by Wilson and Cowan [48], see [49] for a more complete discussion. Although the shape of the synaptic filters does not alter the dynamics of the network, it is a necessary ingredient for the model to generate ING oscillations [49].

Note finally the frequency difference between the PING and the ING rhythm. The two interaction models are then seen as canonical descriptions of the low and fast gamma oscillations, PING for low gamma range and ING for fast gamma spectrum. In both cases, pyramidal cells do not fire in every oscillatory cyle.

Over the past decades, the Phase Resetting Curve (PRC) has become one of the fundamental concepts in theoretical neuroscience. Its usefulness has been reviewed in multiple papers [37–39, 50] and its outcomes are expected to impact our understanding of brain rhythms [27]. PRC measures the effects caused by transient stimuli on oscillatory systems and can be obtained experimentally [51–54].

In our case, the application of a short depolarizing current to the network affects the spiking activity, and the macroscopic oscillation shifts in time, see S1, S2, S3 and S4 Figs. The induced phase shift depends on the perturbation strength but also on the phase at which the perturbation is presented. It can either be delayed or advanced depending on the onset phase of the perturbation. Note that the input can be delivered either to the pyramidal or to the inhibitory neurons in the network.

The PRC results in plotting the advance or delay with respect to the phase onset at which the perturbation is made. Doing so, it quantifies the effect of the perturbation on the macroscopic oscillation. For the cortical network under consideration, several PRCs can reasonably be defined at the same time depending on where the depolarizing input is applied (to the pydamids or the interneurons).

In the limit of short, weak perturbations, the shift in timing can be described by the so-called infinitesimally PRC (iPRC). The iPRC is mathematically expressed by a linear differential system, known as the the adjoint system [55]. This method can be applied to the low dimensional system (2) and (3) and a semi-analytical expression of the macroscopic iPRC be obtained. Assuming that the reduced E-I system (2) and (3) has a stable limit cycle, we find that (see Methods for more detail) the iPRC *Z*(*t*) is a periodic vector that is a solution of the adjoint equation

$$\begin{array}{c} -\frac{d}{dt}Z\left(t\right)=M{\left(t\right)}^{T}\cdot Z\left(t\right), \end{array}$$ (4)

where the matrix M(t) is given by a linearization of the E-I system (2) and (3) around the limit cycle, see Methods for its precise expression.

When the perturbations made to the network are sufficiently small, the PRC becomes proportional to the iPRC [36, 56, 57]. We present in Figs 2E and 3E the iPRC obtained via a simulation of the adjoint system (4) as compared with direct perturbations made on the spiking network. The blue line, (respectively the red line), corresponds to the iPRC of the excitatory input to the I-cells (respectively the E-cells). Note that the noisy aspect of the PRC obtained from the direct method is the consequence of a finite-size effect. The network simulation being made with a finite number of neurons, the firing rate remains somewhat noisy (see Fig 1C and 1D) and the measure of the phase shift is not perfectly accurate. Computing the PRC via the direct method on the reduced system leads to a smoother curve, see S5 Fig.

From the simulations and semi-analytical expression of the PRC we can classify the PING and ING rhythms as having different macroscopic PRC types, i.e. as having different rhythmic properties. For the PING dynamics, see Fig 2E, a biphasic shape of the PRC is observable when perturbations are made on the I-cells. In contrast, when perturbations are on the E-cells, the PRC is monophasic. This is a classification that has already been observed in our previous work where the synaptic dynamics were neglected and considered to be instantaneous [15]. Intuitively this result can be understood as follows: Giving an excitatory pulse to the E-cells, the rhythm can only go faster. On the other hand, a pulse to the I-cells might lead to different effect. If the perturbation is just past the time when the E-cells spike, the rhythm must accelerate, because it helps the I-cells to fire sooner, letting the inhibition wear off sooner and the pyramids can fire sooner on the following cycle. If the perturbation arrives in the middle of the ongoing cycle, it triggers extra I-cell activity which will slow down the rhythm.

Regarding the ING pattern, see Fig 3E, the PRC is monophasic for perturbation targeting the I-cells. The PRC is null when perturbations are made onto the pyramidal cells, which means that any perturbations will die out after a few cycles. This comes without a surprise since in the ING interaction, pyramidal cells do not play a part in the emergence of the oscillations.

PRCs are thus quite different between the ING and the PING oscillations. The difference in shape and type is very robust, and changing the parameters does not affect this observation, see Figs 2F and 3F. This is because the contribution of the cell type to the rhythmic behavior is largely different in the ING and PING mechanisms. The PRC difference between the ING and the PING oscillations has also been noted in a very recent work by Akao and colleagues [35].

From there, we can explore the consequences of differences of locking regimes to periodic pulsatile stimuli, and their result supports that the origin of the cell-type-specific response, already experimentally observed [10], comes from the different entrainment properties [35]. Indeed, biphasic PRCs are known to facilitate entrainment to periodic inputs. This provides some theoretical supports for the implication of inhibitory spiking cells on the locking ability of neural networks.

The amplitudes of the macroscopic PRCs we can also inform us about how sensitive is the network to perturbations onto the excitatory cells versus onto the inhibitory cells. As we see in Figs 2F and 3F, the overall PRC amplitude scaling strongly depends on parameters such as the external current and which cells are targeted by the perturbation. Since a PRC with small amplitude implies that a perturbation will have almost no effect on the oscillatory cycle, the low PRC amplitude can intuitively be interpreted as a stability marker of the oscillations. For instance, the PING oscillation is more sensitive to perturbation to the excitatory cells.

### The phase equation

**Fig 4:**
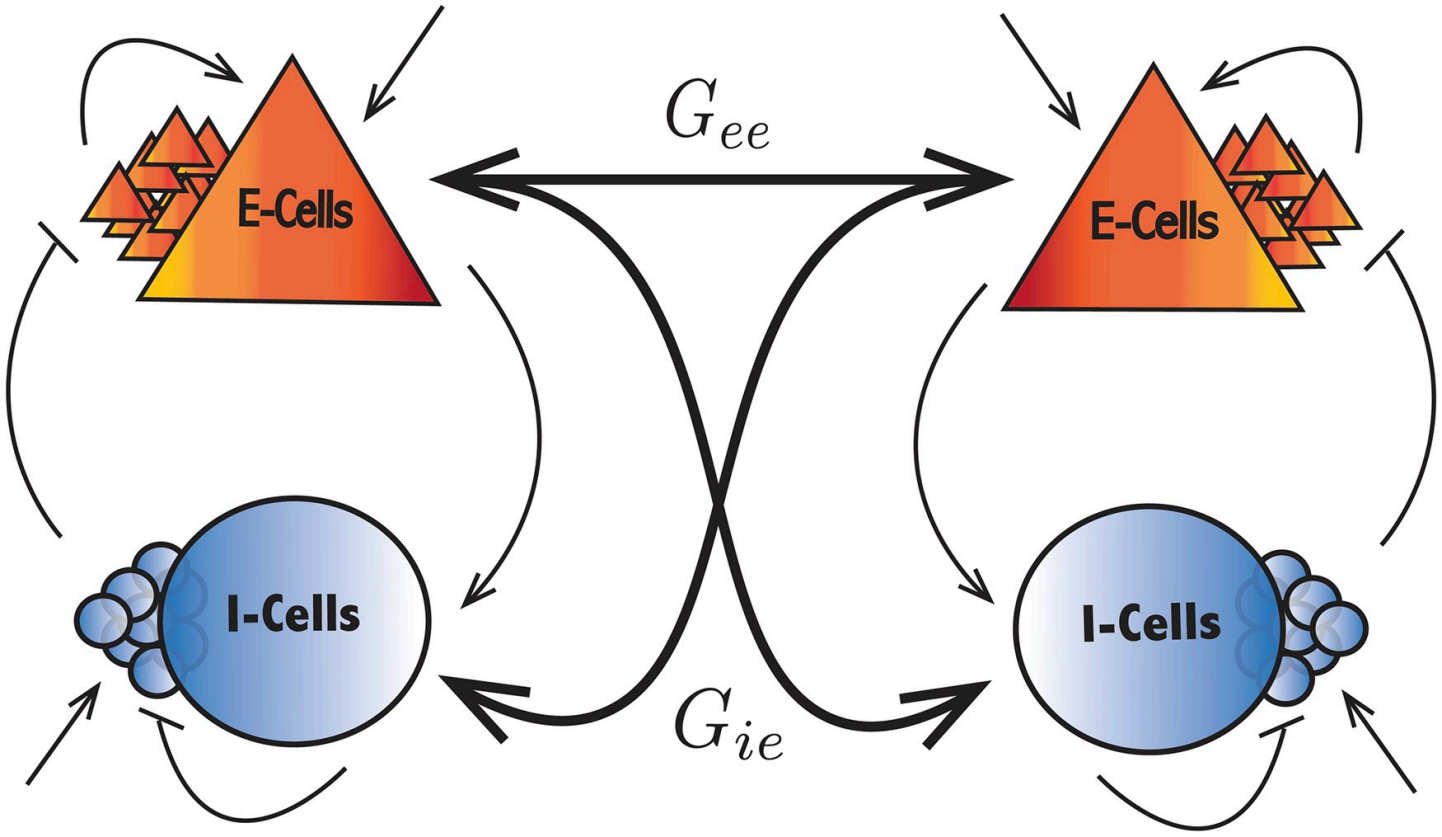
The bidirectionally neural circuits. The connectivity strength of the population *β* of one network onto the population *α* of the other circuit is denoted *G*_*αβ*_. The intrinsic parameters are unchanged and similar within each network as presented in Fig 1.

We now turn to study the dynamical emergence of phase synchrony across multiple networks (as a minimal paradigmatic model reflecting internactions between multiple brain regions). In other words, with the model being minimal, we cannot pretend to aim to study in detail specific brain interactions, however, the structure that is shown in Fig 4 reflects the architecture of many communicating cortical and sub-cortical areas where information transmission is at play [21, 22]. In our set up we consider two coupled spiking circuits. Each circuit is assumed to be made up of interacting pyramidal cells and interneurons as presented in the previous sections (see Fig 1). Since the interneurons are known to make overwhelmingly local connections, the synaptic projection from one circuit to another is made via the pyramidal cells only. A delay, that we treat as a free parameter, is added to account for finite transmission speeds and synaptic time-courses across circuits. Importantly we note that the considered structural motif is symmetric: both circuits are identical and are symmetrically coupled.

While in principle, we could have studied phase locking of circuits showing oscillations at different frequencies, in vivo experimental data suggest that locking across gamma oscillations is most prevalent within the same frequency range [4]. We will thus consider coupled networks with the same frequency and focus our study on two interacting schemes: the PING-PING interaction and the ING-ING interaction. The two mechanistic models of gamma generation having different oscillatory regimes, the interaction PING-ING would lead to a cross-frequency coupling. First, it is far beyond the scope of this paper to investigate the coherence between slow and fast oscillations. Second, we note that, under our knowledge, cross-coupling among slow and fast gamma has not been observed so far.

As one more important point, we note that our whole analysis of phase locked states is based on the assumption that synaptic interactions across the circuits are sufficiently weak. Such an assumption, which guarantees that the perturbed macroscopic oscillations remain close to the unperturbed case, allows us to place our study within the framework of weakly coupled oscillators [39, 40]. We emphasize that within each circuit, neurons are not weakly coupled. The assumption of weak coupling is only made upon the projection from one circuits to another. Within the weakly coupled framework, see Methods, the bidirectionally delayed-coupled neural circuits reduce to a single phase equation:

$$\begin{array}{c} \frac{d}{dt}\theta \left(t\right)=G\left(\theta \left(t\right)\right), \end{array}$$

where *θ*(*t*) is the phase difference (or phase lag) between the circuits and the *G*-function is the odd part of the shifted interaction function, the so-called *H*-function:

$$\begin{array}{c} G(\theta )=H(\theta -d)-H(-\theta -d), \end{array}$$

with *d*, the time delay between the two circuits, and the *H*-function:

$$\begin{array}{c} \begin{array}{cc} H(\theta )= & \frac{G_{ee}}{T}\int_{0}^{T}Z_{s_{ee}}\left(s\right)r_{e}\left(s-\theta \right)\;ds\\ & +\frac{G_{ie}}{T}\int_{0}^{T}Z_{s_{ie}}\left(s\right)r_{e}\left(s-\theta \right)\;ds, \end{array} \end{array}$$

where *T* is the oscillation period and *G*_*αβ*_ denotes the connectivity strength from the population *β* of one circuit onto the population *α* of the other circuit, see Fig 4. Note the involvement of the synaptic component of the PRC *Z*_*s*_(*t*) and the firing rate of the E-cells *r*_*e*_(*t*) all along the oscillatory cycle.

Let us emphasize that the theory used to obtain the functions *H* and *G* is the same than the standard theory used for individual neurons, as it is generic to weakly coupled oscillators. The only difference lies in the coupling, which in our case, is defined via the population firing activity of the excitatory cells. Therefore, the interaction function *H* can be intuitively interpreted as an average effect of the pre-synaptic excitatory firing rate on the phase the second network. The average being computed over one oscillation cycle.

The *G*-function is essential for our study since it conveys knowledge about the possible phase-locking modes between the coupled circuits as well as their stability. Indeed, the zeros of the *G*-function correspond to the steady state phase lags. The stability of a locking mode is conditioned on a negative slope at the zero crossing(s) of this function (*G*′(*θ*) < 0), while a positive slope (*G*′(*θ*) > 0) implies instability, Note that the necessity of a synaptic delay for symmetry breaking and the possibility of switching between symmetry broken leader/follower states have previously been shown in coupled oscillator models [41–44, 58]; however, these results have not previously been shown for spiking neural networks with synaptic delays.

### The inter-circuit locking modes

**Fig 5:**
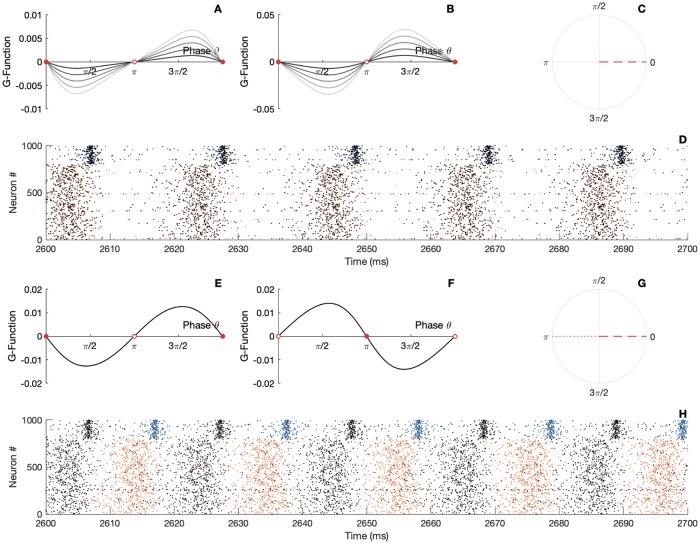
Locking modes of intreacting PING circuits with fixed delay. A) The panel gives the *G*-function for different parameter values *G*_*ee*_ when *G*_*ie*_ = *d* = 0. B) The panel gives the *G*-function for different parameter values *G*_*ie*_ when *G*_*ee*_ = *d* = 0. The circles are filled for stable fixed point and empty for the unstable points. C) Resulting locking mode when there is no delay. D) Raster plot of the spiking activity of the two neural networks, black dots indicate the spike timing of the first network, colored dots indicate the spike timing of the second network, *d* = 0; *G*_*ee*_ = 0.1; *G*_*ie*_ = 0.5. E) The panel gives the *G*-function for *d* = 2, *G*_*ee*_ = 0.1; *G*_*ie*_ = 0.5;. F) The panel gives the *G*-function for *d* = 10; *G*_*ee*_ = 0.1; *G*_*ie*_ = 0.5;. The circles are filled for stable fixed point and empty for the unstable points. G) Resulting locking mode for short and large delay. Note the changes in stability of the modes changes. H) Raster plot of the spiking activity of the two neural networks, black dots indicate the spike timing of the first network, colored dots indicate the spike timing of the second network, *d* = 10; *G*_*ee*_ = 0.1; *G*_*ie*_ = 0.5. Parameters: *N*_*e*_ = *N*_*i*_ = 5000; *τ*_*e*_ = *τ*_*i*_ = 10; *τ*_*se*_ = *τ*_*si*_ = 1; ${\bar{\eta }}_{e}={\bar{\eta }}_{i}=-5$; *J*_*ee*_ = 0; *J*_*ei*_ = 15; *J*_*ii*_ = 0; *J*_*ie*_ = 15; $I_{i}^{ext}=0$; *v*_*th*_ = 500; *v*_*r*_ = −500; $I_{ext}^{e}=10$.

To disentangle the synaptic mechanisms responsible for the dynamical emergence of cross-network phase-locking, we first fix the delay *d* to zero and focus our study on the effect of the coupling strengths. To put it in mathematical terms, we investigate the location of the zeros of the *G*-function with respect to the coupling strengths when the parameter *d* is set to zero. As we see from Fig 5, which show results interacting PING circuits, modifying in the network coupling strength parameters changes the shape of the *G*-function quantitatively, while preserving the phase and the stability of the locked states. The zeros of the *G*-function are located at the in-phase (synchrony) and anti-phase locking (anti-synchrony) mode. The anti-phase state is unstable.

We therefore expect the in-phase synchrony mode to emerge from the dynamics of the bidirectionally coupled circuits. This is the case for a cross-coupling targeting exclusively the E-cells (*G*_*ie*_ = 0, Fig 5A) or the I-cells only (*G*_*ee*_ = 0, Fig 5B). Since in the general case, the interaction function will result in a linear superposition of the two previously mentioned possibilities, in the non-delayed coupling scenario, only a perfect zero lag synchrony can be expected, see Fig 5C. We illustrate this prediction by showing the network rasters in Fig 5D. The black dots correspond to the first network, whereas the colored dots to the second circuit. The spiking activity of the two circuits oscillate in phase, i.e. the two raster plots are synchronized at zero lag and thus overlap. Simulation and theoretical prediction are in perfect agreement. As we can see, despite its vast simplifications, the phase equation yields quantitativley accurate predictions.

The fact that two oscillatory networks (two oscillators) synchronize at zero lag when delay is neglected was to be expected. However, in real settings, neuronal signals travel at finite speeds across the brain and a wide range of delays between neuronal populations has been reported [14]. How the presence of transmission delay reshapes the phase relationship between macroscopic oscillations has remained elusive so far. This is a central issue since recent studies have proposed an updated formulation of the CTC hypothesis where delay between communicating sites plays a critical role [45].

To put it into a mathematical perspective, we expect that distinct delays lead to different fixed-points in the *G*-function, and to illustrate this expectation, we plot the *G*-function obtained for two different example delays (Fig 5E and 5F). As we can see, the stability of the locking modes are reversed, and the anti-phase mode, which was unstable, becomes stable. In contrast, the in-phase mode turns into an unstable state. Two phase-locking modes are then possible: the in phase mode for a short delay and the anti-phase mode for a large delay (Fig 5E and 5F). We illustrate this analytical prediction by showing the network rasters in Fig 5H. As we can see, for large delay value, the spiking activity of the two circuits oscillate in an out of phase mode. Note that for very large values of the delay, the two networks will re-synchronize, see S6 Fig.

**Fig 6:**
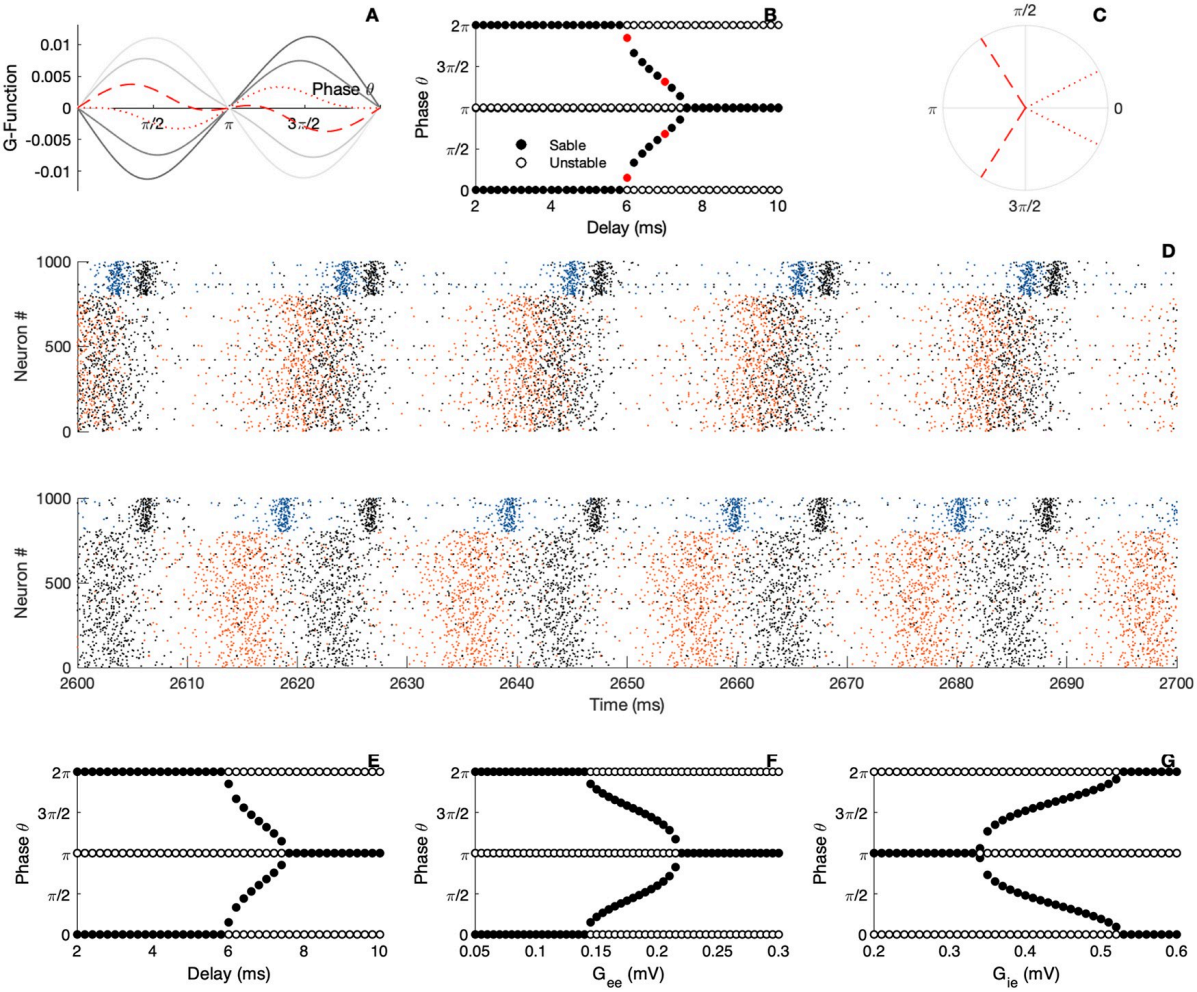
Bifurcation analysis of locking modes with respect to delay for the PING. A) The panel gives the *G*-function for different parameter *d*. B) Bifurcation analysis. The circles are filled for stable fixed point and empty for the unstable points, red dots represent the illustrations parameters C) Resulting locking modes. D) Raster plot of the spiking activity of the two neural networks, black dots indicate the spike timing of the first network, colored dots indicate the spike timing of the second network. E-F-G) Bifurcation analysis. The circles are filled for stable fixed point and empty for the unstable points. panel E) bifurcation parameter *d*, panel F) bifurcation parameter *G*_*ee*_, panel G) bifurcation parameter *G*_*ie*_. Parameters: *N*_*e*_ = *N*_*i*_ = 5000; *τ*_*e*_ = *τ*_*i*_ = 10; *τ*_*se*_ = *τ*_*si*_ = 1; ${\bar{\eta }}_{e}={\bar{\eta }}_{i}=-5$; *J*_*ee*_ = 0; *J*_*ei*_ = 15; *J*_*ii*_ = 0; *J*_*ie*_ = 15; $I_{i}^{ext}=0$; *v*_*th*_ = 500; *v*_*r*_ = −500; $I_{ext}^{e}=10$; *G*_*ee*_ = 0.1; *G*_*ie*_ = 0.5; B) upper panel *d* = 6, lower panel *d* = 7; E) *G*_*ee*_ = 0.1; *G*_*ie*_ = 0.5; F) *G*_*ie*_ = 0.5; *d* = 6; G) *G*_*ee*_ = 0.1; *G*_*ee*_ = 0.1; *d* = 6.

We push our analysis further by investigating the transitions between the two in-phase and anti-phase locking modes we observed above. In Fig 6A we plot the *G*-function obtained for a range of delays. Black lines correspond to small delays while grey lines to bigger ones. A continuous deformation of the coupling function is seen, leading the zeros of the *G*-function to slip over the phase-axis. To get a better visualization, we plot a bifurcation diagram (Fig 6B) which shows us the phase modes positions and stability with respect to parameter change. In the figure, each dot is obtained from the phase at which the G-function intersects the x-axis. It thus displays the phase locations of the zeros of the G-function with respect to the delay. The color black or white indicates the stability of the fixed-point determined from the G-function slope at the zeros Such a diagram helps us to anticipate the locking (or coherent) states in the bidirectionally delayed-coupled networks. We note that the stability of the in-phase mode is kept for small delays. On the other hand, for larger transmission times, a switch of stability between the in-phase and anti-phase locking modes is observed. Importantly, a wide region of delays for which the phase lag goes over all the possibilities appears in the diagram. This result confirms the role of delay in the emergence of a complete variety of phase shifts across gamma interaction in the cortex [17, 59].

In Fig 6D we validate this theoretical prediction by showing rasters of the spiking circuits that reflects the modulation of the emerging phase lag by the delay. As we see from Fig 6D, the spiking activity of the two networks oscillate with a small phase lag. Increasing slightly the delay leads the spiking activity of the two networks to oscillate with a bigger phase lag. Simulation and theoretical prediction are again in perfect agreement. This result shows that it is normal to observe persistent phase relationship across time that are so diverse across brain regions. In general, it is not possible to draw connection between the phase locking diagram (Fig 6B) to the oscillation period. This is only possible when an interaction function is a sine function [40].

As already noticed in [60], this case corresponds to a spontaneous symmetry breaking. We talk about symmetry breaking because those variety of phase lag states do not share the symmetric feature with the full system. Note that when the delay is kept fixed, and sufficiently large, a variation of the synaptic strength onto the E-cells in Fig 6F leads to a transition from the in-phase state to the out-of-phase locking. As a part of this transition, a variety of stable phase lags appear. A reverse situation is depicted in Fig 6G: when the coupling onto the I-cells is varied the in-phase mode transitions to an anti-phase mode. As we can see from these diagrams, we can tune the phase shifts across brain oscillations at least for the PING rhythm.

Of course these result are valid only for weak coupling. When the coupling across the circuits is taken to be too large, the theory will fail in capturing the transition. We also note that the transition between the in phase and the anti-phase modes is still takes place for larger connectivity regime, however it does not happen for values of the delay predicted by the theory, see S7 Fig.

**Fig 7:**
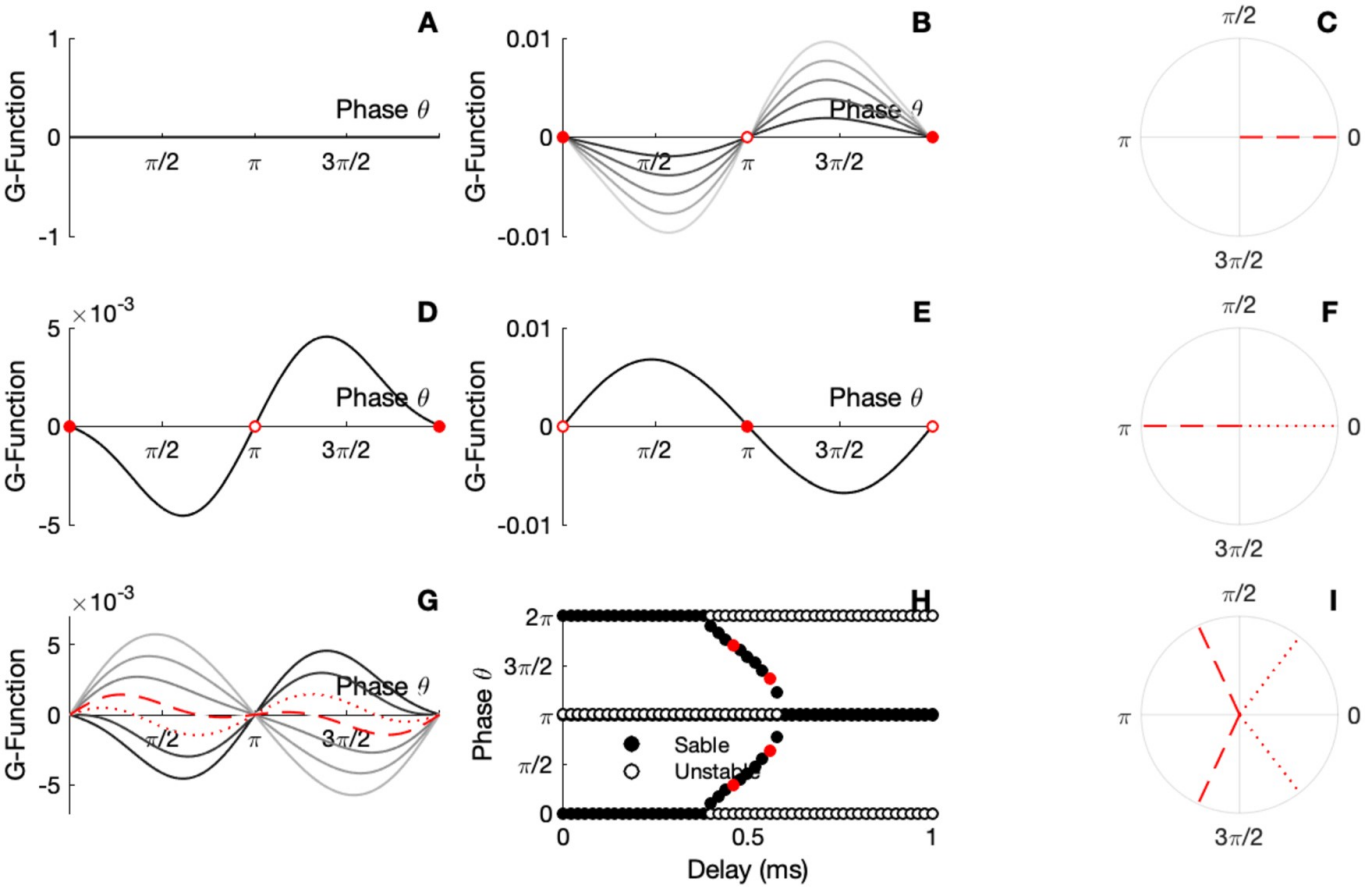
Locking modes of the ING network system. Effect of coupling strength on locking modes without delay. A) The panel gives the *G*-function for different parameter *G*_*ee*_ when *G*_*ie*_ = *d* = 0. B) The panel gives the *G*-function for different parameter *G*_*ie*_ when *G*_*ee*_ = *d* = 0. The circles are filled for stable fixed point and empty for the unstable points. C) Resulting locking mode when there is no delay. D) The panel gives the *G*-function for *d* = 0.1. E) The panel gives the *G*-function for *d* = 0.6. The circles are filled for stable fixed point and empty for the unstable points. F) Resulting locking mode for short and large delay. G) The panel gives the *G*-function for different parameter *d*. H) Bifurcation analysis. The circles are filled for stable and empty for the unstable fixed points, red dots represent the illustrations parameters I) Resulting locking modes. Parameters: *N*_*e*_ = *N*_*i*_ = 5000; *τ*_*e*_ = *τ*_*i*_ = 10; *τ*_*se*_ = *τ*_*si*_ = 1; ${\bar{\eta }}_{e}={\bar{\eta }}_{i}=-5$; *J*_*ee*_ = 0; *J*_*ei*_ = 10; *J*_*ii*_ = 15; *J*_*ie*_ = 0; *v*_*th*_ = 500; *v*_*r*_ = −500; panel A) Δ_*e*_ = Δ_*i*_ = 1; panel B) *I*_*ext*_ = 25; *G*_*ee*_ = 0.; *G*_*ie*_ = 0.3; D) *d* = 0.2, E) *d* = 1.

A similar situation emerges for the ING interaction. In Fig 7, we show the interaction function and corresponding locking modes. While short delays induces only an in-phase locking mode Fig 7C–7F, larger delays will reverse the interaction function and induce an out of phase locking scheme Fig 7E and 7F. Once again, notice the spontaneous symmetry breaking implying the existence of a variety of phase lags for moderate values of the delay Fig 7G, 7H and 7I. Not that for the ING-ING interaction, modification of the synaptic coupling *G*_*αβ*_ will not affect the locking modes since the coupling is through the pyramidal neurons and these do not affect the macroscopic oscillatory phase.

In the above simulations we saw that the two-circuit system can break into a non-symetric dynamic where one network spikes earlier and is followed shortly after within the global firing period. Hence we can call the earlier network the “leader” and the later, the “follower”. We note of course that which networks is the leader and which is the follower, is entirely determined by the network initial condition. In addition, a sufficiently strong transient perturbation to one of the networks, can switch their role, and make the leader a follower and vice versa. This effect can be explained mathematically from the PRC and it has been at the core of recent research on control of the directionality of signal flow [12]. However, making a theory in the case of weakly coupled circuits, we face the difficulty of convergence toward the stable mode. The two networks need to oscillate several cycles in order to reach the fixed point. To speed up the convergence, one would need to increase the coupling which breaks our assumption, see S8 Fig.

### Emerging causal directionality

We now turn to the functional role that could be supported by the dynamic symmetry breaking. Recent studies have associated spontaneous symmetry breaking with an effective transfer of information that is directed [12, 13]. In other words, these works suggested that while the synaptic coupling between networks is fully symmetric, measuring information transfer shows that signals flow prevalently from one network to the other, while it is relatively attenuated in the opposite direction. The conclusion is that despite a symmetric structural connectivity, there is a directed functional connectivity resulting from the on-going network dynamics. However, since most if not all information transfer measures are correlational, functional connectivity has so far been characterized in a statistical manner with a limited implication for causality. We reasoned that our PRC methodology can give us a glimpse at a causal interpretation.

**Fig 8:**
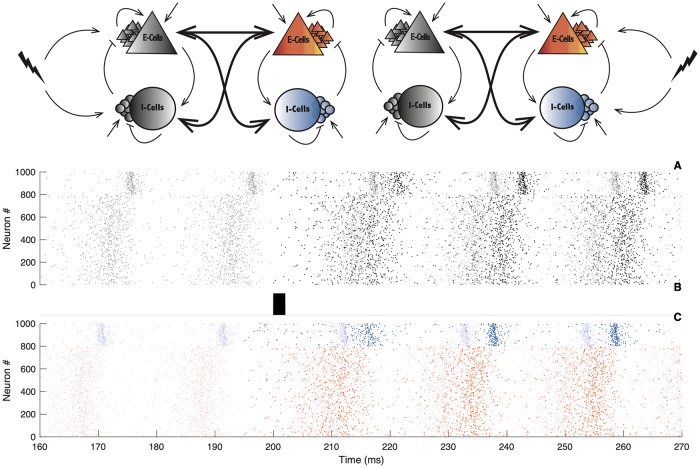
Illustration of the global phase shift. Upper panels: Schematic illustration of the bi-directional coupled spiking neural networks, in grey, the leader, in color, the follower. The leader is defined as spiking early in the global firing cycle, the follower spikes a delay later. The lightning bolt symbolises incoming perturbation on the leader (left panel) or on the follower (right panel). Lower panels: A) Spiking activity of the leader, the transparent spiking activity refers to the unperturbed case, the black dots refer to the perturbed scenario. B) Illustration of the incoming stimulus. C) Spiking activity of the follower, the transparent spiking activity refers to the unperturbed case, the color dots refer the perturbed network. The figure is a cartoon and does not refer to a real simulation. With the presence of the delay across circuits and weak coupling between the circuits, several cycles need to occur before the effect of a perturbation on one of the network can be perceived on the other circuit.

To prove that there is indeed a causal directionality of signaling under symmetry-broken dynamics, we compute the PRC of the full delay system. For that purpose we define a global phase for the whole bi-directionally delayed spiking networks. This is possible because, in a phase-locked state, the spiking activity of the two networks is still periodic. Our intention is to measure how an impact of the input on one of the two networks affects the other circuit and the system as a whole. The logic goes as following; we stimulate one or the other network and measure the global phase shift that results on the two networks. Doing so, we compute what we call a *global PRC*. The global PRC quantifies how the effect of an external perturbation on one network is transferred to the other. In Fig 8 we illustrate this set up. When a short depolarizing current is applied to one network (Fig 8B), the spiking activity and resulting macroscopic oscillation of the two networks will shift in time. A cartoon representing a raster plot illustrates the global phase shift on the spiking activity of the first and second network (Fig 8A–8C). Here the black dots represent the first network, and the colored dots, the second circuit. After the stimulus presentation, spikes are shifted. The global PRC results in plotting this phase shift as a function of the perturbation phase onset. Note that Fig 8 is a cartoon and not a simulation. With the presence of delay across circuits, the phase shift on the second network does not appear as rapidly. We need to wait a few cycles before the effect of a perturbation on one network can be perceived on the other, as the two-circuit system settles to a perturbed firing cycle.

As we pointed out before, in the symmetry broken state, we can heuristically define a leader circuit (one that fires earlier in the global cycle) and a follower circuit (one that fires later). Indeed, the phase difference between the two networks is significantly less than their global period of oscillation. Hence the system fires in a galloping rhythm with one network firing after the other and then a longer delay is apparent before the next volley. We can define that the network firing after the longer period of silence as the leader and the network firing after the subsequent short delay as the follower. We then track how the incoming perturbations (see Fig 8B) to either the leader or the follower shift the spiking activity of both networks (see Fig 8A–8C). We can then see how the global PRC differs when it is obtained from perturbation on the leader or on the follower. We use this difference as a footprint of causal directionality.

While in this manuscript we have sought a fully analytical approach, we find that computing the global PRC is problematic due to the presence of delay. The analytical method’s convergence is not guaranteed. We therefore follow a semi-analytical approach. We use the direct perturbation method to compute the global PRC for the reduced model, which makes the computations efficient (see Method Eqs (9)–(12)). We thus perturb the leader or the follower and observe the resulting asymptotical phase shift of the second network. Of course in the symmetrical dynamical state we expect that the global PRCs of the leader and the follower are identical. We thus posit that should we find that the global PRCs are identical for perturbations to either the leader or the follower, transmission of the incoming perturbation is symmetric. Should the two PRCs differ, we would claim that signal transfer has a directionality.

**Fig 9:**
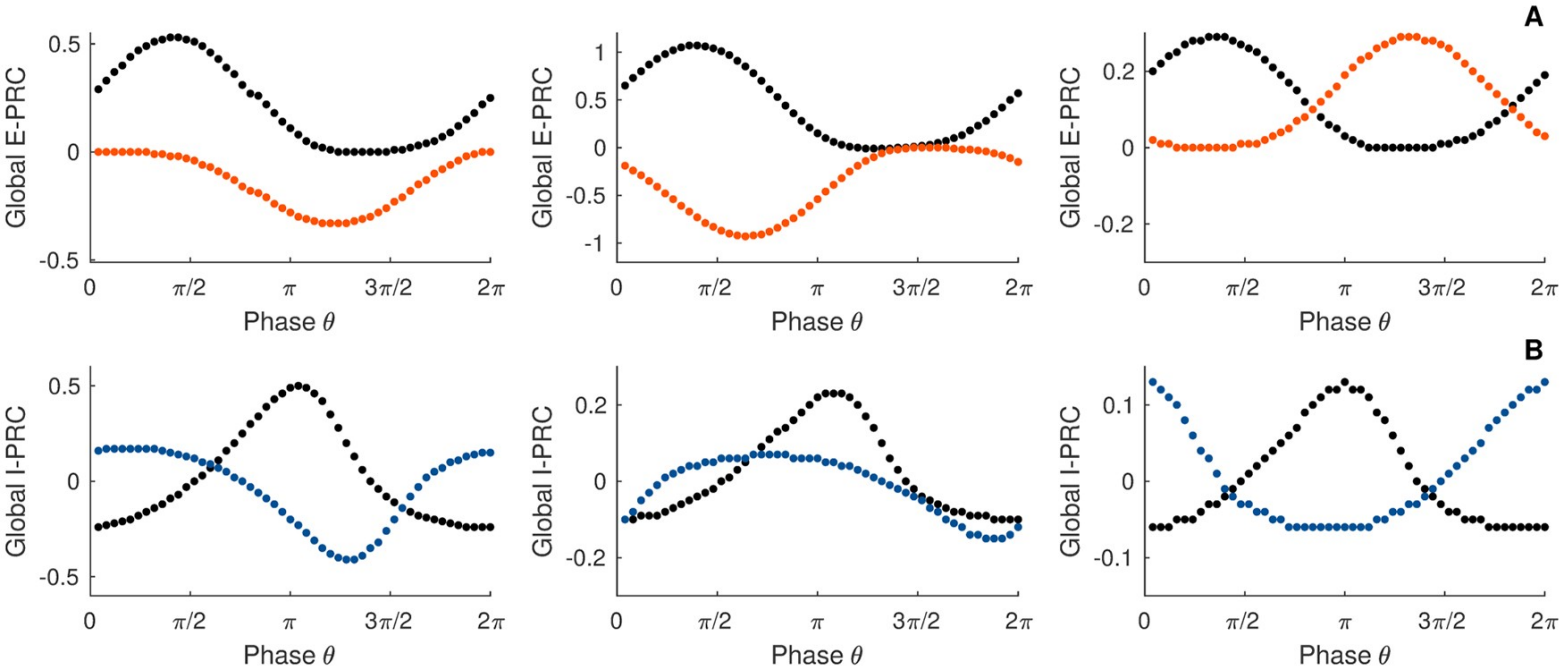
Global PRC and emerging directionality. Black dots illustrate direct perturbations of the leader, color dots indicate direct perturbations of the follower. A) Perturbations are made on the E-cells. B) Perturbations are made on the I-cells. Parameters: *N*_*e*_ = *N*_*i*_ = 5000; *τ*_*e*_ = *τ*_*i*_ = 10; *τ*_*se*_ = *τ*_*si*_ = 1; ${\bar{\eta }}_{e}={\bar{\eta }}_{i}=-5$; *J*_*ee*_ = 0; *J*_*ei*_ = 15; *J*_*ii*_ = 0; *J*_*ie*_ = 15; $I_{i}^{ext}=0$; *v*_*th*_ = 500; *v*_*r*_ = −500; $I_{ext}^{e}=10$; *G*_*ee*_ = 0.1; *G*_*ie*_ = 0.5; direct perturbations are made with a square wave current pulse (amplitude 1, duration 0.1), the first panels *d* = 6, the second panel *d* = 7, the third panel *d* = 10.

Fig 9 illustrates the global PRCs. As expected, when the two networks are in phase, perturbing one or the other has similar outcomes. When the two networks are out of phase, the resulting global PRCs are only shifted with respect to one another. This is a natural consequence of the symmetry in the oscillatory modes of the macroscopic oscillations. The most interesting scenario is when the resulting phase-locking mode is not symmetric. In this situation, perturbing the leader or the follower does not give the same phase shift. As we can see, the leader-evoked and the follower-evoked PRCs are almost reverse, i.e. while a perturbation of the leader induces a phase advance, a perturbation on the follower implies a phase delay. Therefore, our intuition laid out above appears to be supported mathematically by our model. In addition to the intuition above, the amplitudes of the PRCs have also different order of magnitude. Perturbations of the leader have stronger impact than on the follower. Furthermore, we see that for each of the perturbations, phase shifts depend on the phase at which the external “signal” arrives: e.g. there are timings of the input where an excitatory perturbation on either networks advances the oscillations, and timings where perturbing the leader advances the phase, while exciting the follower delays the oscillation.

**Fig 10:**
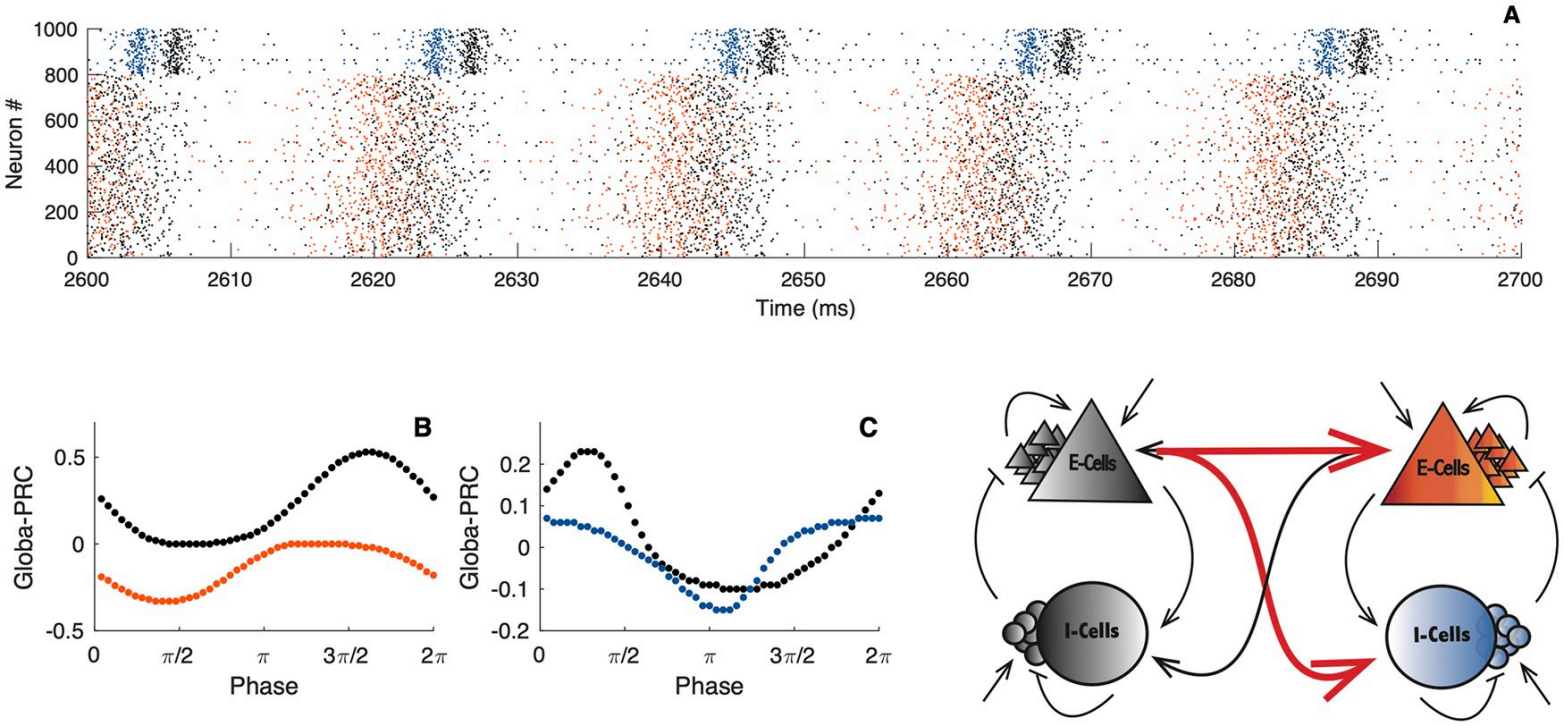
Summary of the resulting directionality. A) Raster plot of the spiking activity of the two networks, black dots indicate the spiking activity of the leader, dots in color, the activity of the follower. B-C) Global PRC obtained from direct perturbation of the leader in black and in the follower in color. In panel B, the perturbation is made on the E-cells, in panel C, the perturbation is made on the I-cells. Lower right panel illustrates the emerging directionality of the signal flow. Parameters: *N*_*e*_ = *N*_*i*_ = 5000; *τ*_*e*_ = *τ*_*i*_ = 10; *τ*_*se*_ = *τ*_*si*_ = 1; ${\bar{\eta }}_{e}={\bar{\eta }}_{i}=-5$; *J*_*ee*_ = 0; *J*_*ei*_ = 15; *J*_*ii*_ = 0; *J*_*ie*_ = 15; $I_{i}^{ext}=0$; *v*_*th*_ = 500; *v*_*r*_ = −500; $I_{ext}^{e}=10$; *G*_*ee*_ = 0.1; *G*_*ie*_ = 0.5; *d* = 6; direct perturbations are made with a square wave current pulse (amplitude 1, duration 0.1).

In summary, we can interpret our results giving a causal directionality in the communication between the two circuits: shifting the phase of the leader has an effect on the follower that is qualitatively different than effect of a follower-phase-shift on the leader. As a note, it has been previously shown that the post-stimulus spike-time histogram (PSTH) can be directly related to the PRC [61, 62]. Hence, the asymmetric PRCs for the leader and the follower predict that the PSTHs tied to perturbing the leader or the follower differ signficantly, once again giving a direct and causal measure of how broken-symmetry states can induce a directional functional connectivity despite complete structural symmetry. In Fig 10 we illustrate a summary of the observation. In the panel Fig 10A, we show the raster plot activity where we can clearly distinguish the leader and the follower, in panels Fig 10B and 10C the corresponding global PRCs, and finally the resulting connectivity of the network in the very last panel. The thick red arrow symbolizes the preference direction of signal flow. This has been recently showed using correlative statistical measures such as transfer entropy [12, 13].

## Discussion

The omnipresence of oscillations in the brain gives significant support to the hypothesis that rhythmic firing patterns are well suited to specific cognitive functions [1, 2]. In particular, recent physiological experiments proposed that coherent gamma rhythms play a determinant part in the transfer of information across cortical areas [7, 9, 45]. As this communication depends on stable phase-relationships between the oscillatory cortical networks, a key question has been to determine the conditions under which two oscillatory brain circuits phase lock, what is the resulting phase lag between them and how the phase lag relates with delay and synaptic couplings [28].

Here, we have outlined and developed a new analytical approach to deal with the dynamical rise of phase synchrony between multiple spiking neural circuits. Making use of a mixture of mathematical techniques—mean-field theory, reduction methods, PRC measures and the framework of weakly coupled oscillators—we have been able to reduce the complexity of the problem to a single phase equation. However, this sequence of mathematical arguments can only be applied to the quadratic integrate-and-fire model when threshold and reset are set at infinity, and assuming a Lorentzian distribution of the bias current [32–34]. Although it does not alter the conclusion, it represents a limitation of our work. Indeed, while similar phase synchronies were observed with conductance-based models such as the Wang-Buzsáki-type conductance-based neurons [12], the line of reasoning to provide a theoretical explanation cannot be reproduced for this type of models.

Let us mention that recently, the macroscopic PRC of an oscillatory network was computed by Akao and colleagues [35]. The main difference with our work is the treatment of noise. In their case, the noise is treated by the use of standard Wiener process mimicking fluctuations of the membrane voltage. In our case, the noise has to be taken into account via a quenched variability expressed only in the form of a Lorentzian probability distribution. However, the continuous nature of the quadratic integrate-and-fire model is required in both approaches to provide an adjoint method [35].

The dynamical phase equation that we obtain using our method fully restitutes the contribution of cortical structure to the coordination of macroscopic firing patterns. More precisely, a nonlinear analysis of the phase equation reveals the role played by the delay and the synaptic coupling across circuits in shaping the locking mode of macroscopic oscillations. We have shown that this level of abstraction suffices to qualitatively reproduce and explain experimentally observed oscillatory patterns. For instance, our synaptic theory allows us to clarify the observed diversity of phase lags between multiple cortical gamma rhythms that have been proposed to play a crucial role in controlling and selecting information through anatomical pathways [17].

Furthermore, our technique allows us to determine the directionality of causal signal transfer between multiple interacting neural circuits with emergent gamma oscillations. Using the PRC technique, we first confirmed that the signal transfer is undirected in dynamical states with full symmetry: the global PRCs were identical or just phase shifted for in-phase and anti-phase synchrony. For dynamical symmetry-broken states, where the circuits separate into a leader and a follower (also sometime called stuttering states), the global PRCs depend qualitatively on where the signal originates (e.g. in the leader) and where it propagates (e.g. to the follower). Our results show that depending on this and on the timing of the external signal perturbations, the neural activity can be either advanced or delayed. Once again, this causal functional directionality in the communication between neural circuits appears as a consequence of the system dynamics and despite a completely mirror symmetric structural connectivity and the individual network properties. We believe that these results give a causal basis for the recent statistical directed functional connectivity measures.

We posit that should we find that the global PRCs are identical for perturbations to either the leader or the follower, transmission of the incoming perturbation is symmetric. Should the two PRCs differ, we would claim that signal transfer has a directionality. For example, should the leader-evoked global PRC be primarily type I and follower-evoked global PRC be type II, one could claim that an excitation to the leader would give an immediate spiking response in the network while exciting the follower would produce a decrease of spiking immediately following the stimulus and hence de facto inhibition (also see [61, 62] for a link between the PRC and the PSTH that supports this intuition). In other words, spikes impinging on the leader would be likely to be transferred by spikes in the network, while spikes impinging on the follower would not.

In the end, the series of mathematical arguments leads to a simple visualization technique—a bifurcation diagram—which compiles all the relevant information about circuit phase relationships when parameters are changed. Such graphical representation demonstrate that, in multiple delayed-coupled spiking networks, phase-locking of the emergent macroscopic oscillatory rhythms are natural features that can be controlled. Our synaptic theory sheds new light on the long range cortical circuit interactions, and importantly, it offers a way to make strong predictions that can be tested against experimental data. For instance, one can compare the phase-locking modes generated by different brain areas with distinct synaptic organization of the model.

The formalism employed within the paper requires pyramidal neurons to work in a regime where projections across circuits are weak. Within this parameter regime, the presented sequence of theoretical arguments are fully valid. How our results extend to the strongly coupled regime remains a challenging topic for future studies.

Although we have restricted our study to considering networks with homogenous synaptic weights and current-based synaptic interaction, the mathematical strategy that served throughout this paper is adjustable and easily accepts the inclusion of conductance-based synaptic description with a certain level of synaptic heterogeneity [34, 63]. Similarly the accommodation of delay within the circuits themselves would not bring difficulty, neither for the reduction method [64], nor for the PRC computation [56]. This could be an interesting subject of research for future works as well as the study of locking to an external periodic modulation for which the PRC offers several path of investigation [35, 65].

All along the paper, we studied locking of oscillations having identical properties, however, several studies have reported coupling across different frequency bands of neural oscillations [59]. Termed as cross-frequency coupling, the locking of brain regions with different frequencies is an open subject of research. A promising extension would then be to generalize our phase-locking analysis to layered network with subsequent layers that include diversified interneuron types along with pyramidal neurons and hence oscillating at different frequencies [66–68]. We project that such analysis would clarify the specific roles of each layer and cell types in the generation of locking and elucidate the underlying synaptic mechanism and functional roles of cross-frequency coupling observed in slow-fast oscillations [59]. Following our PRC framework, we speculate that we would be able to determine the directionality of signalling between such layers. Hence an analytical study of interacting circuits with different intrinsic frequencies remains for us a key open issue to be investigated.

## Methods

### The mean-field

We consider an all-to-all coupled network made up of *N* spiking cells characterized by the quadratic integrate-and-fire (QIF) model:

$$\begin{array}{c} \tau \frac{d}{dt}v_{j}\left(t\right)=\eta_{j}+v_{j}^{2}\left(t\right)+I\left(t\right), \end{array}$$

where *v*(*t*) represents the time evolution of the membrane potential, *τ* is the membrane time constant, *I*(*t*) is the total current, and we assume the intrinsic parameter *η* being randomly distributed across the network according to a Lorentzian distribution:

$$\begin{array}{c} L\left(\eta \right)=\frac{1}{\pi }\frac{\Delta }{{\left(\eta -\bar{\eta }\right)}^{2}+\Delta^{2}}. \end{array}$$

with $\bar{\eta }$ the mean value and Δ the half-width of the distribution. The onset of an action potential is taken into account by a discontinuous mechanism with a threshold *v*_*th*_ and a reset parameter *v*_*r*_ respectively set at plus and minus infinity [30]. The population firing rate is then given by the sum of all the spikes:

$$\begin{array}{c} r\left(t\right)=\frac{1}{N}\sum_{k=1}^{N}\sum_{f}\delta \left(t-t_{f}^{k}\right) \end{array}$$

where *δ* is the Dirac mass measure and $t_{f}^{k}$ are the firing times of the neuron numbered *k*.

In the mean-field limit, that is, when the number of cells is taken infinitely large, see [47] for instance, the system is well represented by the probability of finding the membrane potential of any randomly chosen cell at potential *v* at time *t* knowing the value *η* of its intrinsic parameter. The dynamic of this density, which we denote *p*(*t*, *v*|*η*), is given by a continuous transport equation written in the form of a conservation law:

$$\begin{array}{c} \tau \frac{\partial }{\partial t}p\left(t,v|\eta \right)+\frac{\partial }{\partial v}J\left(t,v|\eta \right)=0, \end{array}$$ (5)

where the total probability flux is defined as

$$\begin{array}{c} J\left(t,v|\eta \right)=(\eta +v^{2}+I\left(t\right))p\left(t,v|\eta \right). \end{array}$$

A boundary condition, consistent with the reset mechanism of the QIF model, is imposed:

$$\begin{array}{c} \lim_{v\to -\infty }J\left(t,v|\eta \right)=\lim_{v\to +\infty }J\left(t,v|\eta \right). \end{array}$$

One can check easily the conservation property of the equation:

$$\begin{array}{c} \int_{-\infty }^{+\infty }p\left(t,v|\eta \right)\;dv=L\left(\eta \right). \end{array}$$

Importantly, the firing rate of the population *r*(*t*) can be extracted from the mean-field equation, defining:

$$\begin{array}{c} r\left(t,\eta \right)=\lim_{v\to +\infty }J\left(t,v|\eta \right), \end{array}$$

the firing rate is then given by the total probability flux crossing the threshold:

$$\begin{array}{c} r\left(t\right)=\lim_{v\to +\infty }\int_{-\infty }^{+\infty }L\left(\eta \right)r\left(t,\eta \right)\;d\eta . \end{array}$$

### Reduction

The reduction method, see [34], consists in assuming that the solution of the mean-field Eq (5) has the form of a Lorentzian distribution:

$$\begin{array}{c} p\left(t,v|\eta \right)=\frac{1}{\pi }\frac{x(t,\eta )}{{\left(v-y\left(t,\eta \right)\right)}^{2}+x{\left(t,\eta \right)}^{2}}. \end{array}$$ (6)

The mean potential and the firing rate are related to the Lorentzian coefficients:

$$\begin{array}{c} r\left(t,\eta \right)=\frac{1}{\pi }x\left(t,\eta \right), \end{array}$$

and

$$\begin{array}{c} y\left(t,\eta \right)=\int_{-\infty }^{+\infty }vp\left(t,v|\eta \right)\;dv. \end{array}$$

Thus the mean membrane potential of the network is

$$\begin{array}{c} V\left(t\right)=\int_{-\infty }^{+\infty }L\left(\eta \right)y\left(t,\eta \right)\;d\eta . \end{array}$$

Note that integrals are defined via the Cauchy principal value, the reason being that the Lorentz distribution only has a mean in the principal value sense. After algebraic manipulation, see [34], the transport Eq (5) reduces to the dynamical system:

$$\begin{array}{c} \left\{ \begin{array}{cc} & \tau \frac{d}{dt}r=\frac{\Delta_{e}}{\pi \tau }+2rV\\ & \tau \frac{d}{dt}V=V^{2}+\bar{\eta }+I-\tau^{2}\pi^{2}r^{2}, \end{array}\right. \end{array}$$

Such a reduced description has the tremendous advantage to be low dimensional.

### E-I interaction

Considering now a network of two interacting neural populations of excitatory cells and inhibitory cells, the system is then represented by two probability density functions, one for the excitatory population, which we denote *p*_*e*_(*t*, *v*|*η*), and one for the inhibitory neurons, which we denote *p*_*i*_(*t*, *v*|*η*). Each density function follows a continuous transport equation similar to (5). In our case, the dynamic of the two coupled PDEs that describe the time evolution of *p*_*e*_(*t*, *v*|*η*) and *p*_*i*_(*t*, *v*|*η*) reduces to a set of differential equations. For the E-cells, we have:

$$\begin{array}{c} \left\{ \begin{array}{cc} & \tau_{e}\frac{d}{dt}r_{e}=\frac{\Delta_{e}}{\pi \tau_{e}}+2r_{e}V_{e}\\ & \tau_{e}\frac{d}{dt}V_{e}=V_{e}^{2}+{\bar{\eta }}_{e}+I_{e}-\tau_{e}^{2}\pi^{2}r_{e}^{2}, \end{array}\right. \end{array}$$

and for the I-cells:

$$\begin{array}{c} \left\{ \begin{array}{cc} & \tau_{i}\frac{d}{dt}r_{i}=\frac{\Delta_{i}}{\pi \tau_{i}}+2r_{i}V_{i}\\ & \tau_{i}\frac{d}{dt}V_{i}=V_{i}^{2}+{\bar{\eta }}_{i}+I_{i}\left(t\right)-\tau_{i}^{2}\pi^{2}r_{i}^{2}. \end{array}\right. \end{array}$$

Note that the two systems are in interaction via the expression of the currents *I*_*e*_ and *I*_*i*_ which include self-recurrent connections and synaptic projections, see Fig 1 for a schematic view. For the E-cells, the total current has the following form:

$$\begin{array}{c} I_{e}\left(t\right)=I_{e}^{ext}\left(t\right)+\tau_{e}s_{ee}\left(t\right)-\tau_{e}s_{ei}\left(t\right), \end{array}$$

and for the I-cells:

$$\begin{array}{c} I_{i}\left(t\right)=I_{i}^{ext}\left(t\right)+\tau_{i}s_{ie}\left(t\right)-\tau_{i}s_{ii}\left(t\right), \end{array}$$

here, *s*_*αβ*_(*t*) represents the time evolution of the synaptic current of the population *β* projected on the population *α* and is given by an exponential filter of the firing activity. In the end, we get that the dynamic of the cortical network is well described by the following set of eight differential equations:

$$\begin{array}{c} \left\{ \begin{array}{cc} & \tau_{e}\frac{d}{dt}r_{e}=\frac{\Delta_{e}}{\pi \tau_{e}}+2r_{e}V_{e}\\ & \tau_{e}\frac{d}{dt}V_{e}=V_{e}^{2}+{\bar{\eta }}_{e}+I_{e}^{ext}+\tau_{e}s_{ee}-\tau_{e}s_{ei}-\tau_{e}^{2}\pi^{2}r_{e}^{2}\\ & \tau_{s}\frac{d}{dt}s_{ee}=-s_{ee}+J_{ee}r_{e}\\ & \tau_{s}\frac{d}{dt}s_{ei}=-s_{ei}+J_{ei}r_{i}, \end{array}\right. \end{array}$$ (7)

and

$$\begin{array}{c} \left\{ \begin{array}{cc} & \tau_{i}\frac{d}{dt}r_{i}=\frac{\Delta_{i}}{\pi \tau_{i}}+2r_{i}V_{i}\\ & \tau_{i}\frac{d}{dt}V_{i}=V_{i}^{2}+{\bar{\eta }}_{i}+I_{i}^{ext}+\tau_{i}s_{ie}-\tau_{i}s_{ii}-\tau_{i}^{2}\pi^{2}r_{i}^{2}\\ & \tau_{s}\frac{d}{dt}s_{ie}=-s_{ie}+J_{ie}r_{e}\\ & \tau_{s}\frac{d}{dt}s_{ii}=-s_{ii}+J_{ii}r_{i}, \end{array}\right. \end{array}$$ (8)

where *τ*_*s*_ is the synaptic time constant, and *J*_*αβ*_ is the synaptic strength of the population *β* projecting on the population *α*.

### Phase response curve

The infinitesimal phase resetting curve (iPRC) is defined mathematically for infinitesimally small perturbation, and it is computed in a perfectly rigorous way via the adjoint method [55]. Let us consider a general dynamical system:

$$\begin{array}{c} \frac{d}{dt}x\left(t\right)=F\left(x\left(t\right)\right), \end{array}$$

where $x\in R^{n}$. Assuming that the system admits a stable limit cycle *x*_0_(*t*), then if the system is perturbed by a small perturbation, the solution can be written as

$$\begin{array}{c} x\left(t\right)=x_{0}\left(t\right)+\epsilon p\left(t\right), \end{array}$$

where *p*(*t*) is the small deviation from the limit cycle. Up to a linearization, we get that

$$\begin{array}{c} \frac{d}{dt}p\left(t\right)=DF\left(x_{0}\left(t\right)\right)\cdot p\left(t\right), \end{array}$$

where *DF*(*x*_0_(*t*)) is the time dependent Jacobian matrix. The iPRC is then defined as

$$\begin{array}{c} \frac{d}{dt}(Z(t)\cdot p(t))=0, \end{array}$$

which is equivalent to

$$\begin{array}{c} \begin{array}{cc} \frac{d}{dt}(Z(t)\cdot p(t)) & =\frac{d}{dt}Z\left(t\right)\cdot p\left(t\right)+Z\left(t\right)\cdot \frac{d}{dt}p\left(t\right)\\ & =\frac{d}{dt}Z\left(t\right)\cdot p\left(t\right)+Z\left(t\right)\cdot DF\left(x_{0}\left(t\right)\right)\cdot p\left(t\right)\\ & =\frac{d}{dt}Z\left(t\right)\cdot p\left(t\right)+DF{\left(x_{0}\left(t\right)\right)}^{T}\cdot Z\left(t\right)\cdot p\left(t\right)\\ & =(\frac{d}{dt}Z\left(t\right)+DF{\left(x_{0}\left(t\right)\right)}^{T}\cdot Z\left(t\right))\cdot p\left(t\right)\\ & =0. \end{array} \end{array}$$

Since the last equation is valid for every perturbation *p*(*t*), we get that the iPRC is solution of the adjoint equation:

$$\begin{array}{c} \frac{d}{dt}Z\left(t\right)=-DF{\left(x_{0}\left(t\right)\right)}^{T}\cdot Z\left(t\right). \end{array}$$

This method can be applied on the low dimensional system (7) and (8) and a semi analytical expression of the iPRC can be extracted. Assuming that

$$\begin{array}{c} O\left(t\right)=(r_{e_{o}}\left(t\right),V_{e_{o}}\left(t\right),s_{ee}\left(t\right),s_{ei}\left(t\right),r_{i_{o}}\left(t\right),V_{i_{o}}\left(t\right),s_{ie}\left(t\right),s_{ii}\left(t\right)), \end{array}$$

is a stable limit cycle of the E-I system (7) and (8) of period *T*, that is,

O(t)=O(t+T)

we find that the iPRC *Z*(*t*) is a periodic vector of eight components

$$\begin{array}{c} Z\left(t\right)=(Z_{r_{e}}\left(t\right),Z_{v_{e}}\left(t\right),Z_{s_{ee}}\left(t\right),Z_{s}{}_{ei}\left(t\right),Z_{r_{i}}\left(t\right),Z_{v_{i}}\left(t\right),Z_{s_{ie}}\left(t\right),Z_{s}{}_{ii}\left(t\right)), \end{array}$$

that is a solution of the adjoint equation

$$\begin{array}{c} -\frac{d}{dt}Z\left(t\right)=M{\left(t\right)}^{T}\cdot Z\left(t\right), \end{array}$$

where the matrix M(t) is given by a linearization of the E-I system (7) and (8) around the limit cycle:

$$\begin{array}{c} M\left(t\right)=[\begin{array}{cccccccc} \frac{2V_{e_{o}}\left(t\right)}{\tau_{e}} & \frac{2r_{e_{o}}\left(t\right)}{\tau_{e}} & 0 & 0 & 0 & 0 & 0 & 0\\ -2\tau_{e}\pi^{2}r_{e_{o}}\left(t\right) & \frac{2V_{e_{o}}\left(t\right)}{\tau_{e}} & 1 & -1 & 0 & 0 & 0 & 0\\ \frac{J_{ee}}{\tau_{s}} & 0 & -\frac{1}{\tau_{s}} & 0 & 0 & 0 & 0 & 0\\ 0 & 0 & 0 & -\frac{1}{\tau_{s}} & \frac{J_{ei}}{\tau_{s}} & 0 & 0 & 0\\ 0 & 0 & 0 & 0 & \frac{2V_{i_{o}}\left(t\right)}{\tau_{i}} & \frac{2r_{i_{o}}\left(t\right)}{\tau_{i}} & 0 & 0\\ 0 & 0 & 0 & 0 & -2\tau_{i}\pi^{2}r_{i_{o}}\left(t\right) & \frac{2V_{i_{o}}\left(t\right)}{\tau_{i}} & 1 & -1\\ \frac{J_{ie}}{\tau_{s}} & 0 & 0 & 0 & 0 & 0 & -\frac{1}{\tau_{s}} & 0\\ 0 & 0 & 0 & 0 & \frac{J_{ii}}{\tau_{s}} & 0 & 0 & -\frac{1}{\tau_{s}} \end{array}]. \end{array}$$

The iPRC *Z*(*t*) is given by the unique periodic solution that satisfies the normalization condition

$$Z\left(t\right)\cdot \dot{O}\left(t\right)=2\pi /T.$$

**Fig 11:**
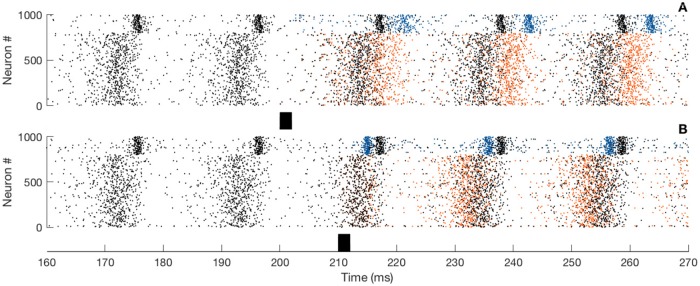
Phase shifting. A-B) Spiking activity obtained from simulations of the full spiking network. The black dots illustrate the ongoing activity and the colored dots (blue for the I-cells and red for the E-cells) the activity of the perturbed network. Lower panels: Illustration of the stimulus onset. Perturbations are made on the I-cells. Parameters: *N*_*e*_ = *N*_*i*_ = 5000; *τ*_*e*_ = *τ*_*i*_ = 10; *τ*_*se*_ = *τ*_*si*_ = 1; ${\bar{\eta }}_{e}={\bar{\eta }}_{i}=-5$; *J*_*ee*_ = 0; *J*_*ei*_ = 15; *J*_*ii*_ = 0; *J*_*ie*_ = 15; $I_{i}^{ext}=0$; *v*_*th*_ = 500; *v*_*r*_ = −500; $I_{ext}^{e}=10$; direct perturbations are made with a square wave current pulse (amplitude 2, duration 2).

The iPRC can be compared with a direct method which consist in presenting perturbation to the network. Depending on the phase onset of the perturbation, the network activity is going to shift. Raster plots from numerical simulations of the full network (Fig 11) illustrate the shift. Here the black dots correspond to the unperturbed network, whereas the colored dots to the perturbed circuit. Before the stimulus onset, the two rasters overlap perfectly. After the stimulus presentation, spikes of the perturbed network are shifted: either delayed (Fig 11A) or advanced (Fig 11B) depending on the onset phase of the perturbation. In the Result section, the two approches—direct perturbation and the adjoint method—are compared for the PING interaction in Fig 2 and the ING interaction in Fig 3.

### Bidirectionally coupled networks

Considering now two bidirectionally delayed coupled networks where the coupling is made via long projections of the pyramidal cells from one network to another, the whole system reduces to a set of sixteen differential equations. For the first network, we have

$$\begin{array}{c} \left\{ \begin{array}{cc} & \tau_{e}\frac{d}{dt}r_{e_{1}}=\frac{\Delta_{e}}{\pi \tau_{e}}+2r_{e_{1}}V_{e_{1}}\\ & \tau_{e}\frac{d}{dt}V_{e_{1}}=V_{e_{1}}^{2}+{\bar{\eta }}_{e}++I_{e}^{ext}+\tau_{e}s_{ee_{1}}-\tau_{e}s_{ei_{1}}-\tau_{e}^{2}\pi^{2}r_{e_{1}}^{2}\\ & \tau_{s}\frac{d}{dt}s_{ee_{1}}=-s_{ee_{1}}+J_{ee}r_{e_{1}}+G_{ee}r_{e_{2}}\left(t-d\right)\\ & \tau_{s}\frac{d}{dt}s_{ei_{1}}=-s_{ei_{1}}+J_{ei}r_{i_{1}}, \end{array}\right. \end{array}$$ (9)

and

$$\begin{array}{c} \left\{ \begin{array}{cc} & \tau_{i}\frac{d}{dt}r_{i_{1}}=\frac{\Delta_{i}}{\pi \tau_{i}}+2r_{i_{1}}V_{i_{1}}\\ & \tau_{i}\frac{d}{dt}V_{i_{1}}=V_{i_{1}}^{2}+{\bar{\eta }}_{i}+I_{i}^{ext}+\tau_{i}s_{ie_{1}}-\tau_{i}s_{ii_{1}}-\tau_{i}^{2}\pi^{2}r_{i_{1}}^{2}\\ & \tau_{s}\frac{d}{dt}s_{ie_{1}}=-s_{ie_{1}}+J_{ie}r_{e_{1}}+G_{ie}r_{e_{2}}\left(t-d\right)\\ & \tau_{s}\frac{d}{dt}s_{ii_{1}}=-s_{ii_{1}}+J_{ii}r_{i_{1}}, \end{array}\right. \end{array}$$ (10)

and for the second network:

$$\begin{array}{c} \left\{ \begin{array}{cc} & \tau_{e}\frac{d}{dt}r_{e_{2}}=\frac{\Delta_{e}}{\pi \tau_{e}}+2r_{e_{2}}V_{e_{2}}\\ & \tau_{e}\frac{d}{dt}V_{e_{2}}=V_{e_{2}}^{2}+{\bar{\eta }}_{e}++I_{e}^{ext}+\tau_{e}s_{ee_{2}}-\tau_{e}s_{ei_{2}}-\tau_{i}^{2}\pi^{2}r_{e_{2}}^{2}\\ & \tau_{s}\frac{d}{dt}s_{ee_{2}}=-s_{ee_{2}}+J_{ee}r_{e_{2}}+G_{ee}r_{e_{1}}\left(t-d\right)\\ & \tau_{s}\frac{d}{dt}s_{ei_{2}}=-s_{ei_{2}}+J_{ei}r_{i_{2}}, \end{array}\right. \end{array}$$ (11)

and

$$\begin{array}{c} \left\{ \begin{array}{cc} & \tau_{i}\frac{d}{dt}r_{i_{2}}=\frac{\Delta_{i}}{\pi \tau_{i}}+2r_{i_{2}}V_{i_{2}}\\ & \tau_{i}\frac{d}{dt}V_{i_{2}}=V_{i_{2}}^{2}+{\bar{\eta }}_{i}+I_{i}^{ext}+\tau_{i}s_{ie_{2}}-\tau_{i}s_{ii_{2}}-\tau_{i}^{2}\pi^{2}r_{i_{2}}^{2}\\ & \tau_{s}\frac{d}{dt}s_{ie_{2}}=-s_{ie_{2}}+J_{ie}r_{e_{2}}+G_{ie}r_{e_{1}}\left(t-d\right)\\ & \tau_{s}\frac{d}{dt}s_{ii_{2}}=-s_{ii_{2}}+J_{ii}r_{i_{2}}, \end{array}\right. \end{array}$$ (12)

Note the presence of long range projections between circuits, see Fig 3 for a schematic view. Here *G*_*αβ*_ denotes the connectivity strength of the population *β* of one network onto the population *α* of the other circuit, and the parameter *d* is the conduction delay between the two networks.

### The phase equation

Assuming that the two networks are oscillating and placing our study within the framework of weakly coupled oscillators, that is, if we assume that

$$G_{\alpha \beta }\ll 1,$$

we can reduce the bidirectionally delayed-coupled neural circuits description (9), (10), (11) and (12) to a single phase equation:

$$\begin{array}{c} \frac{d}{dt}\theta \left(t\right)=G\left(\theta \left(t\right)\right). \end{array}$$

Here *θ*(*t*) is the phase difference (or phase lag) between the circuits and the *G*-function is the odd part of the shifted interaction function (the *H*-function), see [40] for instance:

$$\begin{array}{c} G(\theta )=H(\theta -d)-H(-\theta -d), \end{array}$$

with *d*, the time delay between the two circuits. In our case, the interaction function is mathematically described as

$$\begin{array}{c} \begin{array}{cc} H(\theta )= & \frac{G_{ee}}{T}\int_{0}^{T}Z_{s_{ee}}\left(s\right)r_{e}\left(s-\theta \right)\;ds\\ & +\frac{G_{ie}}{T}\int_{0}^{T}Z_{s_{ie}}\left(s\right)r_{e}\left(s-\theta \right)\;ds, \end{array} \end{array}$$

where *T* is the oscillation period. Note the involvement of the synaptic component of the PRC *Z*_*s*_(*t*) and the firing rate of the E-cells *r*_*e*_(*t*) all along the oscillatory cycle in the expression of the *G*-function.

## Supporting information

S1 FigIllustration of the resynchronization for large delay.A-B-C) Spiking activity obtained from simulations of the full spiking network. The black dots illustrate the spiking activity of the first network and the colored dots the activity of the second network. D) Bifurcation analysis. The circles are filled for stable fixed point and empty for the unstable points, red dots represent the illustrations parameters taken for the panels A-B-C. Parameters: *N*_*e*_ = *N*_*i*_ = 5000; Δ_*e*_ = Δ_*i*_ = 1; *τ*_*e*_ = *τ*_*i*_ = 10; *τ*_*se*_ = *τ*_*si*_ = 1; ${\bar{\eta }}_{e}={\bar{\eta }}_{i}=-5$; *J*_*ee*_ = 0; *J*_*ei*_ = 15; *J*_*ii*_ = 0; *J*_*ie*_ = 15; $I_{e}^{ext}=10$; $I_{i}^{ext}=0$; *v*_*th*_ = 500; *v*_*r*_ = −500; *G*_*ee*_ = 0.1; *G*_*ie*_ = 0.5 with panel A) *d* = 2, panel B) *d* = 12.5, panel C) *d* = 22.(EPS)

S2 FigIllustration of the theory break down for strong coupling.A-B-C-D-E) Spiking activity obtained from simulations of the full spiking network. The black dots illustrate the spiking activity of the first network and the colored dots the activity of the second network. Parameters: *N*_*e*_ = *N*_*i*_ = 5000; Δ_*e*_ = Δ_*i*_ = 1; *τ*_*e*_ = *τ*_*i*_ = 10; *τ*_*se*_ = *τ*_*si*_ = 1; ${\bar{\eta }}_{e}={\bar{\eta }}_{i}=-5$; *J*_*ee*_ = 0; *J*_*ei*_ = 15; *J*_*ii*_ = 0; *J*_*ie*_ = 15; $I_{e}^{ext}=10$; $I_{i}^{ext}=0$; *v*_*th*_ = 500; *v*_*r*_ = −500; *d* = 6 panel A) *G*_*ee*_ = 0.1; *G*_*ie*_ = 0.5, panel B) *G*_*ee*_ = 0.2; *G*_*ie*_ = 1, panel C) *G*_*ee*_ = 0.3; *G*_*ie*_ = 1.5; panel D) *G*_*ee*_ = 0.4; *G*_*ie*_ = 2; panel E) *G*_*ee*_ = 0.5; *G*_*ie*_ = 2.5.(EPS)

S3 FigSpeed convergence to the steady state.A-B-C) Spiking activity obtained from simulations of the full spiking network. The black dots illustrate the ongoing activity and the colored dots (blue for the I-cells and red for the E-cells) the activity of the perturbed network. Parameters: *N*_*e*_ = *N*_*i*_ = 5000; Δ_*e*_ = Δ_*i*_ = 1; *τ*_*e*_ = *τ*_*i*_ = 10; *τ*_*se*_ = *τ*_*si*_ = 1; ${\bar{\eta }}_{e}={\bar{\eta }}_{i}=-5$; *J*_*ee*_ = 0; *J*_*ei*_ = 15; *J*_*ii*_ = 0; *J*_*ie*_ = 15; $I_{e}^{ext}=10$; $I_{i}^{ext}=0$; *v*_*th*_ = 500; *v*_*r*_ = −500; *d* = 6 panel A) *G*_*ee*_ = 0.1; *G*_*ie*_ = 0.5, panel B) *G*_*ee*_ = 0.3; *G*_*ie*_ = 1.5, panel C) *G*_*ee*_ = 0.5; *G*_*ie*_ = 2.5.(EPS)

S4 FigComparison between PRCs.The black line illustrates the analytical adjoint method, and dots indicates direct perturbations of the full spiking neural network. Dots are obtained from perturbation of the full spiking network, or the reduced system. Left panel, the perturbation is delivered to the E-cells. Right panel, the perturbation is delivered to the I-cells. Parameters: *N*_*e*_ = *N*_*i*_ = 5000; *τ*_*e*_ = *τ*_*i*_ = 10; *τ*_*se*_ = *τ*_*si*_ = 1; ${\bar{\eta }}_{e}={\bar{\eta }}_{i}=-5$; *J*_*ee*_ = 0; *J*_*ei*_ = 15; *J*_*ii*_ = 0; *J*_*ie*_ = 15; $I_{i}^{ext}=0$; *v*_*th*_ = 500; *v*_*r*_ = −500; $I_{ext}^{e}=10$; direct perturbations are made with a square wave current pulse (amplitude 5, duration 0.5).(EPS)

S5 FigPhase shifting with perturbation on the I-cells for PING.A) Spiking activity obtained from simulations of the full spiking network and illustration of the stimulus onset. The black dots illustrate the ongoing activity and the colored dots (blue for the I-cells and red for the E-cells) the activity of the perturbed network. Lower panels: Illustration of the stimulus onset. B) Resulting PRC compared with the adjoint method. Parameters: *N*_*e*_ = *N*_*i*_ = 5000; *τ*_*e*_ = *τ*_*i*_ = 10; *τ*_*se*_ = *τ*_*si*_ = 1; ${\bar{\eta }}_{e}={\bar{\eta }}_{i}=-5$; *J*_*ee*_ = 0; *J*_*ei*_ = 15; *J*_*ii*_ = 0; *J*_*ie*_ = 15; $I_{i}^{ext}=0$; *v*_*th*_ = 500; *v*_*r*_ = −500; $I_{ext}^{e}=10$; A) direct perturbations are made with a square wave current pulse (amplitude 10, duration 0.5); B) direct perturbations are made with a square wave current pulse (amplitude 5, duration 0.5).(EPS)

S6 FigPhase shifting with perturbation on the E-cells for PING.A) Spiking activity obtained from simulations of the full spiking network and illustration of the stimulus onset. The black dots illustrate the ongoing activity and the colored dots (blue for the I-cells and red for the E-cells) the activity of the perturbed network. Lower panels: Illustration of the stimulus onset. B) Resulting PRC compared with the adjoint method. Parameters: *N*_*e*_ = *N*_*i*_ = 5000; *τ*_*e*_ = *τ*_*i*_ = 10; *τ*_*se*_ = *τ*_*si*_ = 1; ${\bar{\eta }}_{e}={\bar{\eta }}_{i}=-5$; *J*_*ee*_ = 0; *J*_*ei*_ = 15; *J*_*ii*_ = 0; *J*_*ie*_ = 15; $I_{i}^{ext}=0$; *v*_*th*_ = 500; *v*_*r*_ = −500; $I_{ext}^{e}=10$; A) direct perturbations are made with a square wave current pulse (amplitude 10, duration 0.5); B) direct perturbations are made with a square wave current pulse (amplitude 5, duration 0.5).(EPS)

S7 FigPhase shifting with perturbation on the I-cells for ING.A) Spiking activity obtained from simulations of the full spiking network and illustration of the stimulus onset. The black dots illustrate the ongoing activity and the colored dots (blue for the I-cells and red for the E-cells) the activity of the perturbed network. Lower panels: Illustration of the stimulus onset. B) Resulting PRC compared with the adjoint method. Parameters: *N*_*e*_ = *N*_*i*_ = 5000; *τ*_*e*_ = *τ*_*i*_ = 10; *τ*_*se*_ = *τ*_*si*_ = 1; ${\bar{\eta }}_{e}={\bar{\eta }}_{i}=-5$; *J*_*ee*_ = 0; *J*_*ei*_ = 10; *J*_*ii*_ = 15; *J*_*ie*_ = 0; *I*_*ext*_ = 25; *v*_*th*_ = 500; *v*_*r*_ = −500; A) direct perturbations are made with a square wave current pulse (amplitude 10, duration 0.5); B) direct perturbations are made with a square wave current pulse (amplitude 5, duration 0.5).(EPS)

S8 FigPhase shifting with perturbation on the E-cells for ING.A) Spiking activity obtained from simulations of the full spiking network and illustration of the stimulus onset. The black dots illustrate the ongoing activity and the colored dots (blue for the I-cells and red for the E-cells) the activity of the perturbed network. Lower panels: Illustration of the stimulus onset. B) Resulting PRC compared with the adjoint method. Parameters: *N*_*e*_ = *N*_*i*_ = 5000; *τ*_*e*_ = *τ*_*i*_ = 10; *τ*_*se*_ = *τ*_*si*_ = 1; ${\bar{\eta }}_{e}={\bar{\eta }}_{i}=-5$; *J*_*ee*_ = 0; *J*_*ei*_ = 10; *J*_*ii*_ = 15; *J*_*ie*_ = 0; *I*_*ext*_ = 25; *v*_*th*_ = 500; *v*_*r*_ = −500; A) direct perturbations are made with a square wave current pulse (amplitude 10, duration 0.5); B) direct perturbations are made with a square wave current pulse (amplitude 5, duration 0.5).(EPS)

S9 FigPRC shapes.PRC shape as a function of parameters obtained via the adjoint method for different parameters for the PING. Parameters: *τ*_*e*_ = *τ*_*i*_ = 10; *τ*_*se*_ = *τ*_*si*_ = 1; ${\bar{\eta }}_{e}={\bar{\eta }}_{i}=-5$; *J*_*ee*_ = 0; *J*_*ei*_ = 15; *J*_*ii*_ = 0; *J*_*ie*_ = 15; $I_{i}^{ext}=0$, $I_{e}^{ext}=10$.(EPS)

S10 FigPRC shapes.PRC shape as a function of parameters obtained via the adjoint method for different parameters for the ING. Parameters: *τ*_*e*_ = *τ*_*i*_ = 10; *τ*_*se*_ = *τ*_*si*_ = 1; ${\bar{\eta }}_{e}={\bar{\eta }}_{i}=-5$; *J*_*ee*_ = 0; *J*_*ei*_ = 10; *J*_*ii*_ = 15; *J*_*ie*_ = 0; *I*^*ext*^ = 25.(EPS)

S1 Code The file contains the Matlab code used to make the different figures presented within the manuscript. It also contains the XPPAUT code for the nonlinear analysis.
(ZIP)

## References

[1] Buzsáki G, Draguhn A. Neuronal Oscillations in Cortical Networks. Science. 2004;304(5679):1926–1929. 10.1126/science.1099745 15218136

[2] Buzsáki G. Rhythms of the Brain. Oxford University Press; 2006.

[3] Buzsáki G, Logothetis N, Singer W. Scaling Brain Size, Keeping Timing: Evolutionary Preservation of Brain Rhythms. Neuron;80(3):751–764. 10.1016/j.neuron.2013.10.002 24183025 PMC4009705

[4] Fries P, Nikolia D, Singer W. The gamma cycle. Trends in Neurosciences. 2007;30(7):309–316. 10.1016/j.tins.2007.05.005. 17555828

[5] Buzsáki G, Wang XJ. Mechanisms of Gamma Oscillations. Annual Review of Neuroscience. 2012;35(1):203–225. 10.1146/annurev-neuro-062111-150444 22443509 PMC4049541

[6] Bartos M, Vida I, Jonas P. Synaptic mechanisms of synchronized gamma oscillations in inhibitory interneuron networks. Nat Rev Neurosci. 2007;8(1):45–56. 10.1038/nrn2044 17180162

[7] Fries P. A mechanism for cognitive dynamics: neuronal communication through neuronal coherence. Trends in Cognitive Sciences. 2005;9(10):474–480. 10.1016/j.tics.2005.08.011. 16150631

[8] Strüber M, Sauer JF, Jonas P, Bartos M. Distance-dependent inhibition facilitates focality of gamma oscillations in the dentate gyrus. Nature Communications. 2017;8(1):758 10.1038/s41467-017-00936-3 28970502 PMC5624961

[9] Fries P. Neuronal gamma-band synchronization as a fundamental process in cortical computation. Annual review of neuroscience. 2009;32:209–224. 10.1146/annurev.neuro.051508.135603 19400723

[10] Cardin JA, Carlen M, Meletis K, Knoblich U, Zhang F, Deisseroth K, et al Driving fast-spiking cells induces gamma rhythm and controls sensory responses. Nature. 2009;459(7247):663–667. 10.1038/nature08002 19396156 PMC3655711

[11] Womelsdorf T, Schoffelen JM, Oostenveld R, Singer W, Desimone R, Engel AK, et al Modulation of neuronal interactions through neuronal synchronization. science. 2007;316(5831):1609–1612. 10.1126/science.1139597 17569862

[12] Battaglia D, Witt A, Wolf F, Geisel T. Dynamic Effective Connectivity of Inter-Areal Brain Circuits. PLOS Computational Biology. 2012;8(3):1–20. 10.1371/journal.pcbi.1002438 22457614 PMC3310731

[13] Palmigiano A, Geisel T, Wolf F, Battaglia D. Flexible information routing by transient synchrony. Nature Neuroscience. 2017;20:1014 EP –. 10.1038/nn.4569 28530664

[14] Swadlow HA, Waxman SG. Axonal conduction delays. Scholarpedia. 2012;7.

[15] Dumont G, Ermentrout GB, Gutkin B. Macroscopic phase-resetting curves for spiking neural networks. Phys Rev E. 2017;96:042311 10.1103/PhysRevE.96.042311 29347566

[16] Deco G, Kringelbach ML. Metastability and Coherence: Extending the Communication through Coherence Hypothesis Using A Whole-Brain Computational Perspective. Trends in Neurosciences;39(3):125–135. 10.1016/j.tins.2016.01.001 26833259

[17] Maris E, Fries P, van Ede F. Diverse Phase Relations among Neuronal Rhythms and Their Potential Function. Trends in Neurosciences;39(2):86–99. 10.1016/j.tins.2015.12.004 26778721

[18] Knoblich U, Siegle J, Pritchett D, Moore C. What do We Gain from Gamma? Local Dynamic Gain Modulation Drives Enhanced Efficacy and Efficiency of Signal Transmission. Frontiers in Human Neuroscience. 2010;4:185 10.3389/fnhum.2010.00185 21151350 PMC2981421

[19] Borgers C, Kopell NJ. Gamma Oscillations and Stimulus Selection. Neural Computation. 2007;20(2):383–414. 10.1162/neco.2007.07-06-289 18047409

[20] Borgers C, Epstein S, Kopell NJ. Gamma oscillations mediate stimulus competition and attentional selection in a cortical network model. Proceedings of the National Academy of Sciences. 2008;105(46):18023–18028. 10.1073/pnas.0809511105 PMC2584712 19004759

[21] Buehlmann A, Deco G. Optimal Information Transfer in the Cortex through Synchronization. PLOS Computational Biology. 2010;6(9):1–10. 10.1371/journal.pcbi.1000934 PMC2940722 20862355

[22] Barardi A, Sancristobal B, Garcia-Ojalvo J. Phase-Coherence Transitions and Communication in the Gamma Range between Delay-Coupled Neuronal Populations. PLOS Computational Biology. 2014;10(7):1–17. 10.1371/journal.pcbi.1003723 PMC4110076 25058021

[23] Lowet E, Roberts M, Hadjipapas A, Peter A, van der Eerden J, De Weerd P. Input-Dependent Frequency Modulation of Cortical Gamma Oscillations Shapes Spatial Synchronization and Enables Phase Coding. PLOS Computational Biology. 2015;11(2):1–44. 10.1371/journal.pcbi.1004072 PMC4334551 25679780

[24] Akam T, Kullmann DM. Oscillations and Filtering Networks Support Flexible Routing of Information. Neuron. 2010;67(2):308–320. 10.1016/j.neuron.2010.06.019. 20670837 PMC3125699

[25] Akam TE, Kullmann DM. Efficient “Communication through Coherence” Requires Oscillations Structured to Minimize Interference between Signals. PLOS Computational Biology. 2012;8(11):1–15. 10.1371/journal.pcbi.1002760 23144603 PMC3493486

[26] Akam T, Kullmann DM. Oscillatory multiplexing of population codes for selective communication in the mammalian brain. Nature Reviews Neuroscience. 2014;15:111 EP –. 10.1038/nrn3668 24434912 PMC4724886

[27] Canavier CC. Phase-resetting as a tool of information transmission. Current Opinion in Neurobiology. 2015;31:206–213. 10.1016/j.conb.2014.12.003. 25529003 PMC4375052

[28] Kopell NJ, Gritton HJ, Whittington MA, Kramer MA. Beyond the Connectome: The Dynome. Neuron;83(6):1319–1328. 10.1016/j.neuron.2014.08.016 25233314 PMC4169213

[29] Strogatz SH. From Kuramoto to Crawford: exploring the onset of synchronization in populations of coupled oscillators. Physica D: Nonlinear Phenomena. 2000;143(1):1–20. 10.1016/S0167-2789(00)00094-4.

[30] Izhikevich EM. Dynamical Systems in Neuroscience. Sejnowski TJ, Poggio TA, editors. The MIT Press; 2007.

[31] Ermentrout GB, Terman D. Mathematical foundations of neuroscience. Springer; 2010.

[32] Ott E, Antonsen TM. Low dimensional behavior of large systems of globally coupled oscillators. Chaos. 2008;18(3). 10.1063/1.2930766. 19045487

[33] Luke TB, Barreto E, So P. Complete Classification of the Macroscopic Behavior of a Heterogeneous Network of Theta Neurons. Neural Computation. 2013;25(12):3207–3234. 10.1162/NECO_a_00525 24047318

[34] Montbrió E, Pazó D, Roxin A. Macroscopic Description for Networks of Spiking Neurons. Phys Rev X. 2015;5:021028.

[35] Akao A, Ogawa Y, Jimbo Y, Ermentrout GB, Kotani K. Relationship between the mechanisms of gamma rhythm generation and the magnitude of the macroscopic phase response function in a population of excitatory and inhibitory modified quadratic integrate-and-fire neurons. Phys Rev E. 2018;97:012209 10.1103/PhysRevE.97.012209 29448391

[36] Kotani K, Yamaguchi I, Yoshida L, Jimbo Y, Ermentrout GB. Population dynamics of the modified theta model: macroscopic phase reduction and bifurcation analysis link microscopic neuronal interactions to macroscopic gamma oscillation. Journal of The Royal Society Interface. 2014;11(95). 10.1098/rsif.2014.0058 PMC4006245 24647906

[37] Stiefel KM, Ermentrout GB. NEURONS AS OSCILLATORS. Journal of Neurophysiology. 2016;. 10.1152/jn.00525.2015 27683887 PMC5192043

[38] Ashwin P, Coombes S, Nicks R. Mathematical Frameworks for Oscillatory Network Dynamics in Neuroscience. The Journal of Mathematical Neuroscience. 2016;6(1):2 10.1186/s13408-015-0033-6 26739133 PMC4703605

[39] Nakao H. Phase reduction approach to synchronisation of nonlinear oscillators. Contemporary Physics. 2016;57(2):188–214. 10.1080/00107514.2015.1094987

[40] Ermentrout B, Ko TW. Delays and weakly coupled neuronal oscillators. Philosophical Transactions of the Royal Society of London A: Mathematical, Physical and Engineering Sciences. 2009;367(1891):1097–1115. 10.1098/rsta.2008.0259 19218153

[41] Woodman MM, Canavier CC. Effects of conduction delays on the existence and stability of one to one phase locking between two pulse-coupled oscillators. Journal of Computational Neuroscience. 2011;31(2):401–418. 10.1007/s10827-011-0315-2 21344300 PMC3130804

[42] Wang S, Chandrasekaran L, Fernandez FR, White JA, Canavier CC. Short Conduction Delays Cause Inhibition Rather than Excitation to Favor Synchrony in Hybrid Neuronal Networks of the Entorhinal Cortex. PLOS Computational Biology. 2012;8(1):1–19. 10.1371/journal.pcbi.1002306 22241969 PMC3252263

[43] Ermentrout GB, Kopell N. Fine structure of neural spiking and synchronization in the presence of conduction delays. Proceedings of the National Academy of Sciences. 1998;95(3):1259–1264. 10.1073/pnas.95.3.1259 PMC18738 9448319

[44] Zeitler M, Daffertshofer A, Gielen CCAM. Asymmetry in pulse-coupled oscillators with delay. Phys Rev E. 2009;79:065203 10.1103/PhysRevE.79.065203 19658549

[45] Fries P. Rhythms for Cognition: Communication through Coherence. Neuron. 2015;88(1):220–235. 10.1016/j.neuron.2015.09.034. 26447583 PMC4605134

[46] Ermentrout B. Ermentrout-Kopell canonical model. 2008;3(3):1398.

[47] Deco G, Jirsa VK, Robinson PA, Breakspear M, Friston K. The Dynamic Brain: From Spiking Neurons to Neural Masses and Cortical Fields. PLoS Comput Biol. 2008;4(8):1–35. 10.1371/journal.pcbi.1000092 PMC2519166 18769680

[48] Wilson HR, Cowan JD. Excitatory and Inhibitory Interactions in Localized Populations of Model Neurons. Biophysical Journal;12(1):1–24. 10.1016/S0006-3495(72)86068-5 4332108 PMC1484078

[49] Devalle F, Roxin A, Montbrió E. Firing rate equations require a spike synchrony mechanism to correctly describe fast oscillations in inhibitory networks. ArXiv e-prints. 2017;. 10.1371/journal.pcbi.1005881 PMC5764488 29287081

[50] Smeal RM, Ermentrout GB, White JA. Phase-response curves and synchronized neural networks. Philosophical Transactions of the Royal Society of London B: Biological Sciences. 2010;365(1551):2407–2422. 10.1098/rstb.2009.0292 20603361 PMC2894948

[51] Reyes AD, Fetz EE. Two modes of interspike interval shortening by brief transient depolarizations in cat neocortical neurons. Journal of Neurophysiology. 1993;69(5):1661–1672. 10.1152/jn.1993.69.5.1661 8389834

[52] Akam T, Oren I, Mantoan L, Ferenczi E, Kullmann DM. Oscillatory dynamics in the hippocampus support dentate gyrus-CA3 coupling. Nat Neurosci. 2012;15(5):763–768. 10.1038/nn.3081 22466505 PMC3378654

[53] Stiefel KM, Gutkin BS, Sejnowski TJ. The effects of cholinergic neuromodulation on neuronal phase-response curves of modeled cortical neurons. Journal of computational neuroscience. 2009;26(2):289–301. 10.1007/s10827-008-0111-9 18784991 PMC2857973

[54] Phoka E, Cuntz H, Roth A, Hausser M. A New Approach for Determining Phase Response Curves Reveals that Purkinje Cells Can Act as Perfect Integrators. PLOS Computational Biology. 2010;6(4):1–14. 10.1371/journal.pcbi.1000768 PMC2861707 20442875

[55] Brown E, Moehlis J, Holmes P. On the Phase Reduction and Response Dynamics of Neural Oscillator Populations. Neural Computation. 2004;16(4):673–715. 10.1162/089976604322860668 15025826

[56] Kotani K, Yamaguchi I, Ogawa Y, Jimbo Y, Nakao H, Ermentrout GB. Adjoint Method Provides Phase Response Functions for Delay-Induced Oscillations. Phys Rev Lett. 2012;109:044101 10.1103/PhysRevLett.109.044101 23006090

[57] Nakao H, Yanagita T, Kawamura Y. Phase-Reduction Approach to Synchronization of Spatiotemporal Rhythms in Reaction-Diffusion Systems. Phys Rev X. 2014;4:021032.

[58] Borgers C. An Introduction to Modeling Neuronal Dynamics. 2017;66 10.1007/978-3-319-51171-9

[59] Canolty RT, Knight RT. The functional role of cross-frequency coupling. Trends in Cognitive Sciences. 2010;14(11):506–515. 10.1016/j.tics.2010.09.001 20932795 PMC3359652

[60] Battaglia D, Brunel N, Hansel D. Temporal Decorrelation of Collective Oscillations in Neural Networks with Local Inhibition and Long-Range Excitation. Phys Rev Lett. 2007;99:238106 10.1103/PhysRevLett.99.238106 18233419

[61] Gutkin BS, Ermentrout GB, Reyes AD. Phase-Response Curves Give the Responses of Neurons to Transient Inputs. Journal of Neurophysiology. 2005;94(2):1623–1635. 10.1152/jn.00359.2004 15829595

[62] Ermentrout GB, Galan RF, Urban NN. Relating Neural Dynamics to Neural Coding. Phys Rev Lett. 2007;99:248103 10.1103/PhysRevLett.99.248103 18233494 PMC2533709

[63] Ratas I, Pyragas K. Macroscopic self-oscillations and aging transition in a network of synaptically coupled quadratic integrate-and-fire neurons. Phys Rev E. 2016;94:032215 10.1103/PhysRevE.94.032215 27739712

[64] Pazó D, Montbrió E. From Quasiperiodic Partial Synchronization to Collective Chaos in Populations of Inhibitory Neurons with Delay. Phys Rev Lett. 2016;116:238101 10.1103/PhysRevLett.116.238101 27341262

[65] Kuhn T, Helias M. Locking of correlated neural activity to ongoing oscillations. PLOS Computational Biology. 2017;13(6):1–32. 10.1371/journal.pcbi.1005534 PMC5484611 28604771

[66] Jiang X, Shen S, Cadwell CR, Berens P, Sinz F, Ecker AS, et al Principles of connectivity among morphologically defined cell types in adult neocortex. Science. 2015;350 (6264). 10.1126/science.aac9462 PMC4809866 26612957

[67] Potjans TC, Diesmann M. The Cell-Type Specific Cortical Microcircuit: Relating Structure and Activity in a Full-Scale Spiking Network Model. Cerebral Cortex. 2014;24(3):785–806. 10.1093/cercor/bhs358 23203991 PMC3920768

[68] Bos H, Diesmann M, Helias M. Identifying Anatomical Origins of Coexisting Oscillations in the Cortical Microcircuit. PLOS Computational Biology. 2016;12(10):1–34. 10.1371/journal.pcbi.1005132 PMC5063581 27736873

